# Comparative analysis of DNA methylome and transcriptome of skeletal muscle in lean-, obese-, and mini-type pigs

**DOI:** 10.1038/srep39883

**Published:** 2017-01-03

**Authors:** Yalan Yang, Guoming Liang, Guanglin Niu, Yuanyuan Zhang, Rong Zhou, Yanfang Wang, Yulian Mu, Zhonglin Tang, Kui Li

**Affiliations:** 1 State Key Laboratory of Animal Nutrition, Institute of Animal Science, Chinese Academy of Agricultural Sciences, Beijing 100193, China; 2 Agricultural Genome Institute at Shenzhen, Chinese Academy of Agricultural Sciences, Shenzhen, 518124, China

## Abstract

DNA methylation plays a pivotal role in biological processes by affecting gene expression. However, how DNA methylation mediates phenotype difference of skeletal muscle between lean-, obese-, and mini-type pigs remains unclear. We systematically carried out comparative analysis of skeletal muscle by integrating analysis of genome-wide DNA methylation, mRNA, lncRNA and miRNA profiles in three different pig breeds (obese-type Tongcheng, lean-type Landrace, and mini-type Wuzhishan pigs). We found that the differentially methylated genes (DMGs) were significantly associated with lipid metabolism, oxidative stress and muscle development. Among the identified DMGs, 253 genes were related to body-size and obesity. A set of lncRNAs and mRNAs including *UCP3, FHL1, ANK1, HDAC4,* and *HDAC5* exhibited inversely changed DNA methylation and expression level; these genes were associated with oxidation reduction, fatty acid metabolism and cell proliferation. Gene regulatory networks involved in phenotypic variation of skeletal muscle were related to lipid metabolism, cellular movement, skeletal muscle development, and the p38 MAPK signaling pathway. DNA methylation potentially influences the propensity for obesity and body size by affecting gene expression in skeletal muscle. Our findings provide an abundant information of epigenome and transcriptome that will be useful for animal breeding and biomedical research.

The domestic pig (*Sus scrofa*), an economically important farm animal, is widely used in biomedical research and comparative genome studies, because of physiological (the organs of the pig are similar in size to those of humans), metabolic, and genomic similarities with humans^1^,^2^,^3^,^4^. Long-term artificial selection and intensive breeding programs have resulted in considerable phenotypic diversity of skeletal muscle in domestic pig, which promote domestic pig as an ideal model organism for studies aimed at understanding the molecular mechanisms of muscle development, human stature, obesity, and muscle-related diseases. Skeletal muscle is the most abundant tissue in the human body and is highly metabolically active^5^,^6^. Skeletal muscle is highly heterogeneous, it is composed of fibers with different morphological, contractile and metabolic characteristics including slow-oxidative (type I and IIa) and fast-twitch glycolytic (type IIb) fibers^7^. Muscle mass is determined by the number of muscle fibers and their size. Variations in muscle fiber type composition influence energy metabolism, glucose utilization, muscular endurance and animal meat quality^8^,^9^,^10^. Significant differences in meat quality and muscle mass, which are affected by muscle fiber characteristics and intramuscular fat content, exist among pig breeds^11^,^12^. For example, in comparison with lean-type breeds (e.g., western commercial breeds such as Yorkshire and Landrace (LD)), obese-type breeds (e.g., Chinese indigenous pigs breeds such as Tongcheng (TC), Meishan and Lantang) have more type I and less type IIb fibers, smaller muscle fiber diameter and fewer myofibers^13^,^14^; moreover, obese-type breeds exhibit greater intramuscular fat content^15^,^16^. In contrast, miniature pigs (e.g., Chinese native Wuzhishan (WZS) and Bama pigs) have much less muscle mass and growth rate than obese- and lean-type pigs^17^. A comprehensive understanding of the genetic basis of skeletal muscle performance is crucial for animal breeding and human biomedical research related to muscle.

Transcriptome analysis of prenatal skeletal muscle revealed that the gene expression profiles of TC and LD pigs differed significantly. In particular, TC pigs exhibited a slower muscle growth rate and more complicated molecular changes, while the expression levels of genes associated with increasing cellular growth and myoblast survival were comparatively up-regulated in LD pigs^18^. Recently, our group integrated miRNA and mRNA expression profiles of prenatal and adult skeletal muscle from TC, LD, and WZS pigs, revealing that the myoblast proliferation phases of LD and TC pigs were longer than that of WZS pigs^19^,^20^. Zhao *et al*. found that the myogenic regulatory factors and myocyte enhancer factor 2 gene families are critical mediators of phenotypic differences between Lantang and LD pigs; moreover, in comparison with LD pigs, myogenesis was initiated earlier, but progressed more slowly in Lantang pigs^14^. Another study showed that muscle fiber formation in TC pigs began earlier than muscle fiber formation in Yorkshire pigs, while the muscle maturation process was comparatively more complicated in young Yorkshire piglets^13^. These studies have illuminated the genetic basis of the phenotype of skeletal muscle and identified candidate genes related to muscle development. However, transcriptome information alone cannot fully explain the molecular mechanisms underlying phenotypic variation observed in skeletal muscle.

Epigenetic factors (e.g. miRNA, lncRNA, and DNA methylation) regulate gene expression and serve as key regulators of skeletal muscle development, metabolism, and other processes^21^,^22^,^23^,^24^. Genome-wide analyses of miRNAs and lncRNAs have been performed to explore phenotypic variation of skeletal muscle in pigs^19^,^20^,^25^,^26^,^27^. Several miRNAs and lncRNAs associated with muscle development have been identified, including miRNA-1/206, miRNA-133, miRNA-155, miRNA-127, miRNA-148a, Malat1 and H19^28^,^29^,^30^,^31^,^32^. DNA methylation is an epigenetic modification in which a methyl group is covalently attached to the fifth carbon of cytosine residues^33^. DNA methylation in the promoter region and gene body can stably alter gene expression; this process is an important influence on developmental and tissue-specific gene expression^34^,^35^.

In comparison other tissue types, skeletal muscle exhibits a specific DNA methylation signature^36^,^37^. Hypermethylation is generally abolished during myogenic terminal differentiation and hypomethylation has very strong associations with genes related to contractile fiber^38^. Several studies have been performed to explore the genome-wide methylation profiling in pig muscle and other tissues using reduced representation bisulfite sequencing (RRBS)^39^,^40^ or methylated DNA immunoprecipitation sequencing (MeDIP-seq) methods^41^,^42^. Jin *et al*. reported that genes involved in aging processes exhibited gene body hypomethylation in the *longissimus dorsi* muscle in middle-aged pigs in comparison with the young pigs^41^. Li *et al*. carried out genome-wide DNA methylation profiling in adipose and skeletal muscle tissues from three pig breeds, revealing that differentially methylated regions in promoters were highly associated with obesity development via repressing expression of known obesity-related genes and novel genes^42^. However, the mechanisms through which epigenetic regulation influence muscle performance in mammals remain largely unclear; the mechanisms underlying phenotypic variation in skeletal muscle may be revealed by assessing genome-wide DNA methylation signatures together with the transcriptome (including mRNA, lncRNA and miRNA), allowing candidate genes to be identified and assessed in future studies.

In this study, we performed DNA methylome profiling of porcine skeletal muscle in obese-type TC, lean-type LD, and mini-type WZS pigs using MeDIP-seq. Integrated analysis of genome-wide DNA methylation and transcriptome was performed to reveal the manner in which DNA methylation may regulate muscle performance by affecting gene expression. We also constructed miRNA-mRNA-methylation interaction networks to illuminate the potential epigenetic mechanisms determining the phenotype of skeletal muscle in the domestic pig. Our study provides an empirical basis for further development of the domestic pig as a model organism for studies aimed at understanding human growth, obesity, and muscle-related diseases.

## Results

### Overview of genome-wide DNA methylation profiling

**Figure 1:**
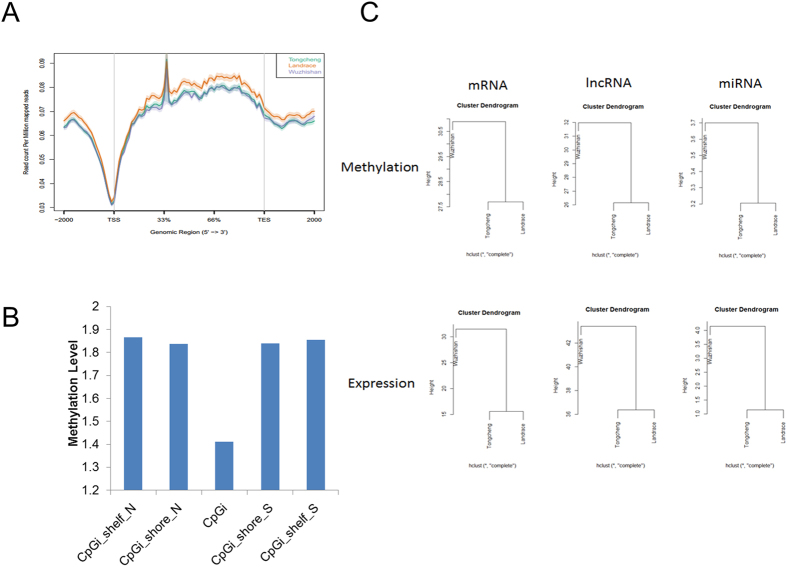
Genome-wide methylation distribution in different genomic elements. (**A**) Methylation distribution of gene body and flanking regions. The gene body was defined as the region from TSS to TTS. In gene body, each gene was split into 60 equal windows. In upstream and downstream 2 kb regions, the regions were split into 20 non-overlap windows. Reads per million mapped reads values was calculated for each window. (**B**) Methylation level around the CpG islands. The methylation level of different genomic elements was calculated by RPKM method. (**C**) Clustering analysis of the DNA methylation and expression profiles of mRNAs, lncRNAs, and miRNAs in the skeletal muscle of TC, LD, and WZS pigs.

In order to understand the effects of DNA methylation on phenotypic variation of skeletal muscle, we firstly carried out MeDIP-seq analysis in skeletal muscle from obese-type TC, lean-type LD, and mini-type WZS pigs. After removing the contaminated, low quality and adaptors sequences, approximately 61 million clean reads were obtained for each library. Quality control checks suggested that the clean sequences were of high quality (Figure S1). Analysis of TC, LD, and WZS pigs showed that 83.82%, 85.52%, and 84.36%, respectively, of the clean sequences were mapped to the *Sus scrofa* reference genome (version 10.2). Saturation analysis showed that the sequencing depth of the MeDIP-seq data was sufficient for further study (Figure S2). Coverage analysis revealed that approximately 80% of all CpGs in the *Sus scrofa* genome were covered at least one-fold, whereas nearly 50% of CpGs were covered more than 5-fold (Figure S3). Next, we evaluated methylation profiles around gene body regions. As expected, a similar pattern of canonical genic DNA methylation was observed among the selected pig breeds. Generally, the DNA methylation pattern around the transcription start site (TSS) showed a V-shaped curve in all three breeds, after a sharp increase in methylation in the 5′-region of the gene body, the methylation level remained relatively constant until the transcription ending site (TES), whereas hypomethylation was observed at the 3′-ends of genes (Fig. 1A). These findings were in concordance with previous reports regarding pigs^39^,^43^,^44^. CpG islands (CpGi) showed significantly reduced methylation in comparison with that of CpGi shores or CpGi shelfs (Fig. 1B). We performed correlation analysis of the DNA methylome in different genomic elements across breeds, revealing a strong positive correlation (r = 0.75–0.94, all P-values are less than 2.2e-16) across breeds in all genomic elements (Figure S4). The 5′- and 3′-untranslated regions (UTRs) exhibited lower correlation coefficients in comparison with those of other regions, indicating low conservation of methylation in UTR regions across breeds. Intronic regions exhibited stronger correlations across breeds in comparison with those of other intragenic elements. Moreover, in comparison with CpGi shores and CpGi shelfs, CpGi methylation was less conserved across breeds (Figure S4).

### Comparison of DNA methylation among breeds

Clustering analysis of the DNA methylation profiles of mRNAs, lncRNAs, and miRNAs showed that TC and LD pigs were clustered together first, after which they were clustered with WZS pigs (Fig. 1C), indicating that the DNA methylation statuses of TC and LD pigs were more similar to each other than to that of WZS pigs. The DNA methylation profiles between breeds are consistent with their expression profiles of mRNAs, lncRNAs, and miRNAs (Fig. 1C). These findings suggest that DNA methylation might contribute to the gene expression profiles of TC, LD, and WZS pigs.

**Figure 2:**
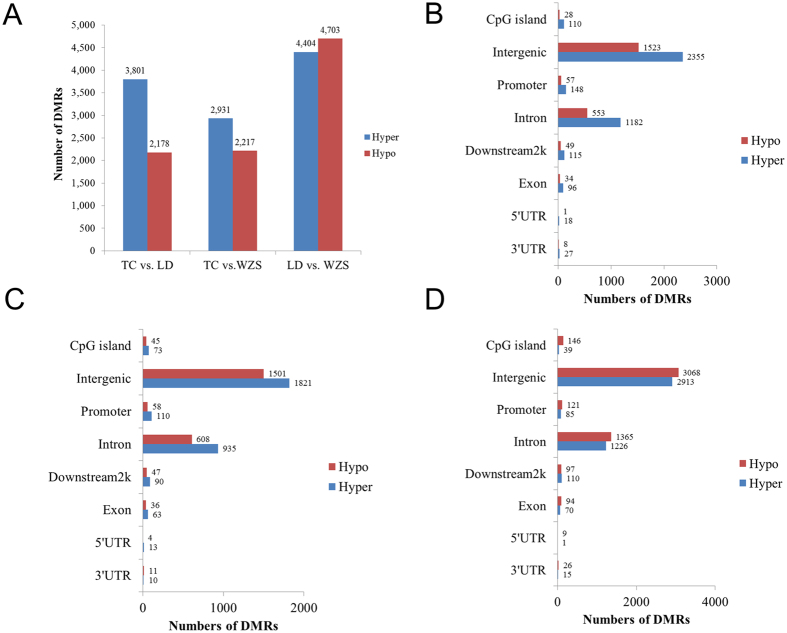
Identification of differentially methylated regions among the TC, LD and WZS pigs. (**A**) The number of differentially methylated regions between different pig breeds. Histograms showing the distribution numbers of DMRs in different genomic elements in TC vs. LD group (**B**), TC vs. LD group (**C**), LD vs. WZS group (**D**).

To further compare the DNA methylation profiles of the selected breeds, the MEDIPS package^45^ was used to identify differentially methylated regions (DMRs) (FDR < 0.05) across breeds. After merging significant neighboring windows, we detected 5,979 DMRs (3,801 hypermethylated and 2,178 hypomethylated regions), 5,148 DMRs (2,931 hypermethylated and 2,217 hypomethylated regions), and 9,107 DMRs (4,404 hypermethylated and 4,703 hypomethylated regions), respectively, in the TC vs. LD, TC vs. WZS, and LD vs. WZS comparisons (Fig. 2A, detailed list of DMRs in Tables S1–S3). The LD vs. WZS comparison yielded more DMRs than were produced by the other comparisons, indicating that the WZS and LD groups had the greatest differences in DNA methylation patterns among the selected breeds. Interestingly, our transcriptome analysis of skeletal muscle identified more differentially expressed mRNAs and miRNAs between the LD and WZS breeds than were identified in the TC vs. LD and TC vs. WZS comparisons, whereas the LD and TC breeds had the most similar gene expression profiles among the three breeds^19^,^46^. These findings suggest that mini-type and lean-type pigs are less closely related than either breed is to obese-type pigs. Notably, comparison analysis of DMRs between breeds showed that both LD and WZS pigs had more hypomethylated regions than hypermethylated regions when compared with TC pigs (Fig. 2A). The distribution of DMRs showed that the majority of DMRs were located at intergenic and intron elements (Fig. 2B–D).

### Validation of MeDIP-seq data by bisulfite sequencing

**Figure 3:**
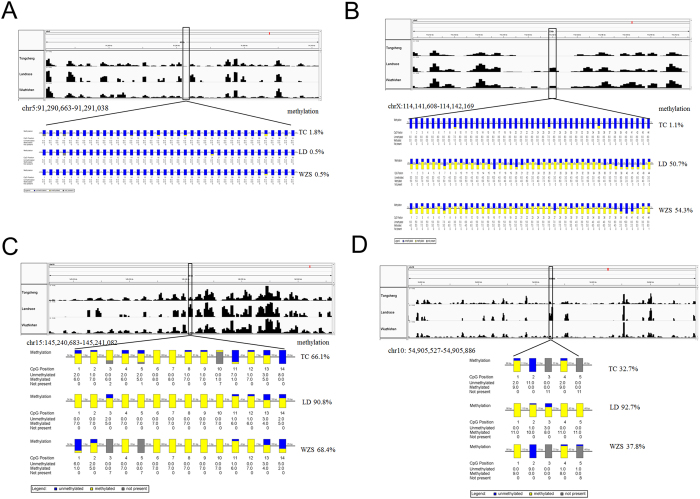
Validation of MeDIP-seq data by bisulfite sequencing PCR. (**A**) A low methylated region across the TC, LD, and WZS pigs in the promoter of the ENSSSCG00000000890 gene on Chromosome 5 from 91,290,663 to 91,291,043. (**B**) A differentially methylated region on Chromosome X from 114,141,608 to 114,142,169. (**C**) A differentially methylated region across the three pig breeds on Chromosome 15 from 145,240,683 to 145,241,082. (**D**) A differentially methylated region across the three pig breeds on Chromosome 10 from 54,905,527 to 54,905,886. The visualization of DNA methylation signal of MeDIP-seq along the chromosome in each pig breed were performed by Integrative Genomics Viewer (top panels). The bottom panels show the results of bisulfite sequencing PCR. Each box corresponds to one CpG position in the genomic sequence. The colored bars summarize the methylation states of all sequences at that position, blue indicates unmethylated and yellow indicates methylated.

We randomly selected ten regions to confirm the reliability of the MeDIP-seq data by bisulfite sequencing PCR (BSP) with additional skeletal muscle samples. These regions included one region with a relatively high level of methylation in the *RHBDF1* gene (Figure S5), one region with a moderate level of methylation in the *COL5A1* gene (Figure S6), one region with a low level of methylation in the promoter of the ENSSSCG00000000890 gene across all three pig breeds (Fig. 3A), and 6 DMRs between different pig breeds (Fig. 3B–D, Figures S7–S10). The methylation differences of several DMRs (Figures S7–S10) validated by BSP were smaller than the results of MeDIP-seq, but the trend tended to be quite consistent, these differences could be explained by the methylation variation across different individuals within breeds. Overall, the results of bisulfite sequencing PCR were in good agreement with our MeDIP-seq data, indicating that the methylation data from MeDIP-seq were reliable for further study.

### Differentially methylated genes and function annotation

**Figure 4:**
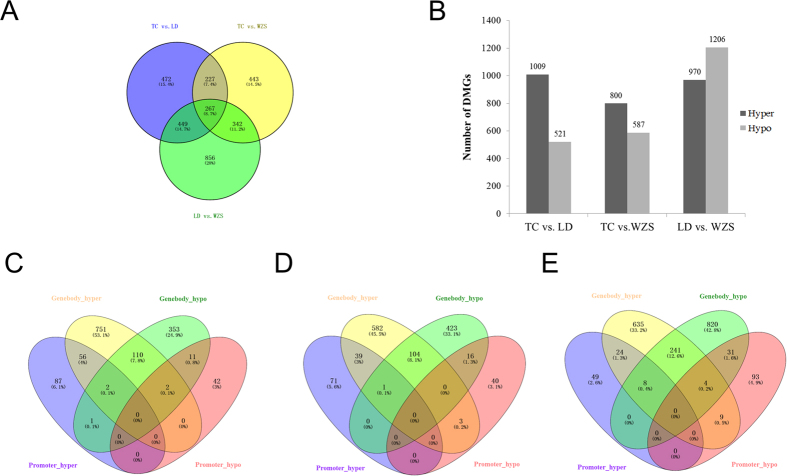
Differentially methylated genes among the TC, LD, and WZS pigs. (**A**) Differentially Methylated genes that were unique or shared among three groups of TC, LD, and WZS pigs. (**B**) The number of hypermethylated and hypomethylated genes among different pig breeds. Venn diagram of the numbers of DMGs in promoter and gene-body in TC vs. LD group (**C**), TC vs. WZS group (**D**), LD vs. WZS group (**E**).

We mapped DMRs to genes based on their genomic location, a total of 3,054 differentially methylated genes were identified (Fig. 4A). Of these, 1,415 genes (corresponding to 1,009 hypermethylated and 521 hypomethylated genes), 1,279 genes (corresponding to 587 hypermethylated and 800 hypomethylated genes) and 1,914 genes (corresponding to 970 hypermethylated and 1,206 hypomethylated genes), respectively, were differentially methylated in the TC vs. LD, TC vs. WZS, and LD vs. WZS comparisons (Fig. 4B). Gene ontology (GO) and KEGG analyses were performed to evaluate the functions of DMGs in skeletal muscle. In the TC vs. LD group, DMGs were involved in positive regulation of GTPase activity, regulation of signal transduction, regulation of cell communication, and developmental process (FDR < 0.05, Table S4). KEGG pathway analysis revealed these DMGs were significantly enriched in glycine, serine and threonine metabolism, histidine metabolism, and other metabolism related pathways. In the TC vs. WZS group, DMGs were significantly associated with localization, developmental cell growth, cell morphogenesis, and enzyme linked receptor protein signaling pathway (FDR < 0.05, Table S5). KEGG pathway analysis revealed that these DMGs were significantly involved in axon guidance, B cell receptor signaling, phenylalanine metabolism, biosynthesis of unsaturated fatty acids, MAPK signaling, and focal adhesion. In the LC vs. WZS group, DMGs were significantly enriched in biological processes related to phosphorus metabolic process, lipid metabolic process, positive regulation of signal transduction, and cell morphogenesis involved in differentiation (FDR < 0.05, Table S6). KEGG pathway analysis revealed that these DMGs were significantly associated with biosynthesis of unsaturated fatty acids, glycerolipid metabolism, PPAR signaling, and MAPK signaling.

Genes that exhibited DMRs in their promoters and gene bodies are shown in Fig. 4C–E. The TC vs. LD, TC vs. WZS, and LC vs. WZS comparisons contained 115, 108, and 262 genes, respectively, containing both hypermethylated and hypomethylated regions. Moreover, 267 DMGs were common to all three sets of DMGs (Fig. 4A). GO analysis suggested that the common genes were mainly involved in regulation of GTPase activity, apoptosis, cell cycle, and cell death (Figure S11), whereas KEGG analysis revealed that these genes were involved in histidine metabolism and axon guidance. These findings suggest that the methylation status of DMGs significantly contributes to phenotypic variation of skeletal muscle across different pig breeds.

### Differential methylation of genes associated with body size and obesity

There are significant differences in body size, muscle mass and obese index among the TC, LD and WZS pigs. Genome-wide association studies (GWASs) for height, weight, obesity and body mass index (BMI) in humans provide a good basis for investigating muscle phenotype variation in pigs. We interrogated the identified DMGs against the catalogue of published GWASs in GWASdb V2^47^, revealing 253 DMGs that were orthologous to human genes related to body-size and obesity (Table S7). For example, insulin-like growth factor I receptor (*IGF1R)*, which plays a critical role in skeletal muscle development and differentiation^48^, was hypermethylated in LD pigs compared with TC and WZS pigs. The methylation level of the *IGF1R* promoter was increased in diabetic mice compared with normal mice; this change was accompanied by a decrease in its mRNA level^49^. It is associated with human height traits and contributed to reduced size in dogs^50^; moreover, it is an important candidate gene for postnatal growth and carcass composition traits in pigs^51^. Genes related to body size and obesity were significantly enriched (Fisher’s exact test, p-value = 3.23e-12). In addition, skeletal muscle metabolic genes (e.g. *HDAC4, HDAC5*, and *UCP3*) were also found in the list. These findings indicate that altered methylation of genes associated with body size and obesity significantly contributes to phenotypic variation of skeletal muscle in mammals. In addition, these findings show that the domestic pig is a suitable model for human biomedical research, especially for studies of growth, metabolism, and obesity-related diseases.

### Global correlation analysis between DNA methylation and mRNA expression

**Figure 5:**
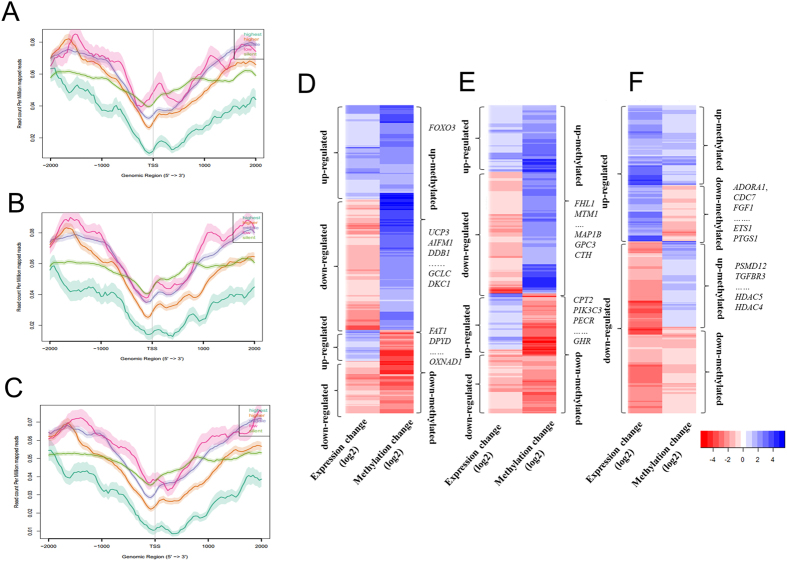
Integrative analysis of DNA methylome and transcriptome in the skeletal muscle of TC, LD, and WZS pigs. DNA methylation level distributions around TSS of five levels of gene expression in the TC (**A**), LD (**B**), and WZS (**C**) pigs. Heatmap of methylation and expression change of DMRs and corresponding genes in the TC vs. LD group (**D**), the TC vs. WZS group (**E**), and the LD vs. WZS group (**F**).

It’s well known that methylation in promoter regions and gene body can regulate gene expression^34^,^35^. Our recent transcriptome study explored differences in gene expression in skeletal muscle from LD, TC, and WZS pigs^20^. To determine the regulatory potential of DMGs, we integrated methylome and transcriptome to assess the effects of DNA methylation on gene expression in skeletal muscle phenotypic variation. The DNA methylation profiles around the TSS regions of genes with five levels of expression were evaluated. The expressed genes showed a methylation pattern with a typical V-shape curve at the TSS, while silently expressed genes did not show this pattern. A significant negative correlation was observed between DNA methylation status around the TSS and gene expression (Fig. 5A–C). These findings are in agreement with previous observations that the methylation density of highest expressed genes was lowest at their TSS and remained low even downstream of the TSS^52^. However, the methylation pattern of silently expressed genes around TSS was clearly different from those of the expressed genes, suggesting that other epigenetic factors might be associated with gene silencing. Although the relationship between gene-body DNA methylation and expression levels showed a non-monotonic correlation, the gene body of highest expressed genes was much lowly DNA methylated compared with other genes (Figure S12).

In the TC vs. LD, TC vs. WZS, and LD vs. WZS groups, we found 377, 338, and 604 unique intragenic DMRs, respectively, associated with genes that were differentially expressed by at least 1.5-fold (Fig. 5D–F). Many genes contained more than one DMR in their intragenic regions. For example, four DMRs in *ANK1* were hypermethylated in WZS pigs compared with TC pigs. In the TC vs. LD group, we identified 112 hypermethylated genes with down-regulated expression in LD pigs, including genes related to fatty acid metabolism and lipid biosynthesis (e.g., *ECHDC2, FAR1, UCP3, UGCG*, and *ST6GALNAC6*), cell proliferation (e.g., *PSPH, GPC4*, and *DKC1*), and response to stress (e.g., *RAD21, AIFM1, DDB1*, and *GCLC*), while 31 genes were hypomethylated and up-regulated in LD pigs compared with TC pigs, including *FAT1*, which has large effects on fatness and growth^53^ (Fig. 5D). Moreover, we found eight genes related to oxidation reduction (*BLVRA, FAR1, ALDH7A1, AIFM1, MAOA, MICAL3, OXNAD1,* and *DPYD*) exhibiting opposite trends in DNA methylation and gene expression in the TC vs. LD group. In the TC vs. WZS group, 99 genes were hypermethylated and down-regulated in WZS pigs, these genes were significantly related to regulation of growth (e.g., *MAP1B, GPC3, CTH*, and *HTRA1*), cell proliferation (e.g., *GAB1, CSGALNACT1, GPC4*, and *INSIG1*) and muscle development (e.g., *FHL1* and *MTM1*). In contrast, 49 genes were hypermethylated and down-regulated in TC pigs, these genes markedly related to lipid modification and fatty acid metabolism (e.g., *CPT2, PIK3C3, HSD17B4, PECR*, and *GHR*) (Fig. 5E). In LD vs. WZS group, 100 genes were hypermethylated and down-regulated in WZS pigs, including genes related to cell proliferation (e.g. *ADORA1, CDC7, FGF1, PTGS1*, and *ETS1*), lipid transport and biosynthetic (e.g. *OSBPL10, SLC27A1, FADS2*, and *PTGS1*), and muscle contraction (e.g. *GUCY1A3* and *PTGS1*). We found that 133 genes were hypomethylated and up-regulated in WZS pigs, these genes were mainly related to cell cycle (e.g., *APBB2, BRCA1, MNAT1, PSMD12*, and *SART1*), cell size (e.g*., CD38, APBB2*, and *TGFBR3*) and muscle hypertrophy and metabolism (e.g., *HDAC5* and *HDAC4*) (Fig. 5F). In addition, some genes exhibited coinciding methylation and expression patterns among breeds (Fig. 5D–F). For example, *FOXO3,* a transcription factor which controls autophagy in skeletal muscle^54^ and involves in muscle energy homeostasis^55^, was intronic hypermethylated and up-regulated in Landrace pigs compared with Tongcheng pigs. These findings suggest that the mechanisms regulating gene expression are complex and involve many elements, including transcription factors, miRNAs, lncRNAs, and circRNAs.

### DNA methylation and expression signatures of lncRNAs

**Figure 6:**
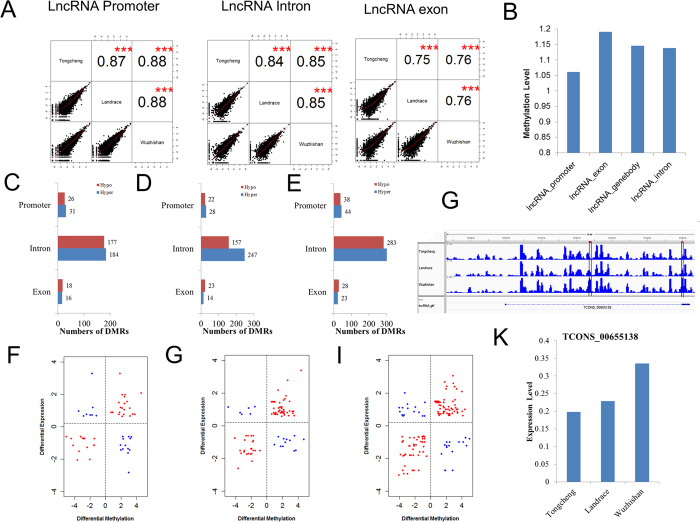
DNA Methylation and expression signatures of lncRNAs in the skeletal muscle of TC, LD, and WZS pigs. (**A**) Correlation analyses across pig breeds in the promoter, exon, and intron regions of lncRNAs. (**B**) Methylation level in different lncRNA elements. Distribution of differentially methylated regions in different elements in TC vs. LD group (**C**), TC vs. LD group (**D**), LD vs. WZS group (**E**). Scatter plot of methylation and expression change of DMRs and corresponding lncRNAs in TC vs. LD group (**F**), TC vs. LD group (**G**), LD vs. WZS group (**H**). (**I**) The methylate level of a region in TCONS_00655138 was higher in WZS pigs than that in the TC and LD pigs. (**J**) The expression level of TCONS_00655138 in the WZS pigs was much higher than that in the TC and LD pigs.

Long noncoding RNAs (lncRNAs) play a critical role in many developmental processes, including myoblast differentiation^56^, adipogenesis^57^, cell cycle^58^, and cell proliferation^59^. Thousands of lncRNAs have been identified in pigs and other mammals^27^,^57^,^60^,^61^,^62^. DNA methylation modulates expression of long intergenic noncoding RNAs in porcine adipose and muscle tissues^60^. Recently, we carried out a genome-wide analysis of lncRNAs across tissues and developmental skeletal muscle, and resulting in the identification of 10,813 lncRNAs in pigs (paper under review). In this study, we observed the DNA methylation status of these lncRNAs and identified differentially methylated lncRNAs in skeletal muscle among different breed pigs. First, we examined correlations among the lncRNA methylation profiles of different breeds. We found that methylation of lncRNAs had high correlation between breeds and the correlation coefficient ranged from 0.75 to 0.88 at different lncRNA elements (all *P*-values are less than 2.2e-16, Fig. 6A). The correlation coefficients across breeds at promoter and intron region were higher than at exons (Fig. 6A). The average methylation level of lncRNAs at exons was higher than those of lncRNAs at promoters and introns (Fig. 6B), consistent with previous studies^63^,^64^. We identified 452, 491, and 853 DMRs in lncRNAs in the TC vs. LD, TC vs. WZS, and LC vs. WZS groups, respectively. It is notable that most DMRs were found in intronic regions (Fig. 6C–E). We further mapped DMRs identified to annotated lncRNAs according to their genomic location. We detected 436 differentially methylated lncRNAs between the TC and LD pigs, of which 249 lncRNAs were hypermethylated in LD pigs, while 212 lncRNAs were hypermethylated in TC pigs (Table S8). We identified 413 differentially methylated lncRNAs between the TC and WZS pigs, including 204 hypermethylated lncRNAs and 233 hypomethylated lncRNAs in WZS pigs (Table S9). In the LD vs. WZS group, we detected 657 differentially methylated lncRNAs (corresponding to 358 hypermethylated lncRNAs and 361 hypomethylated lncRNAs in WZS pigs) (Table S10). Subsequently, we performed GO enrichment analysis using protein-coding genes neighboring differentially methylated lncRNAs. No biological processes were significantly enriched in the TC vs. LD group. In the TC vs. WZS group, neighboring genes were significantly enriched in terms related to anti-apoptosis, cell death, neuron differentiation, and DNA repair (FDR < 0.05) (Figure S13A). In the LD vs. WZS group, neighboring genes were significantly associated with regulation of apoptosis, cell death, and homeostatic process (FDR < 0.05) (Figure S13B).

To explore whether methylation affects lncRNA expression, we identified 5,595 expressed lncRNAs in skeletal muscle (Table S11). 64, 69, and 102 lncRNAs were significantly differently expressed in the TC vs. LD, TC vs. WZS, and LD vs. WZS groups, respectively (log2|FC| ≥ 1, FDR ≤ 0.05) (Tables S12–14). Subsequently, we detected DMR-lncRNA pairs in the three comparisons. The DMR-lncRNA pairs were defined as lncRNAs that showed an expression change of at least 1.5-fold and were differentially methylated in the gene body or promoter regions across breeds. The analysis revealed that 57, 95, and 152 DMR-lncRNA pairs were identified in the TC vs. LD, TC vs. WZS, and LD vs. WZS groups, respectively (Fig. 6F–H). The number of DMR-lncRNA pairs with a positive correlation was greater than the number of pairs with a negative correlation in all three comparisons.

In the TC vs. LD group, 12 lncRNAs were significantly hypermethylated and down-regulated, while 8 lncRNAs were significantly hypomethylated and up-regulated in LD pigs (Fig. 6F). In the TC vs. WZS group, 10 lncRNAs were significantly hypermethylated and down-regulated in WZS pigs. Moreover, 6 lncRNAs were significantly hypomethylated and up-regulated in WZS pigs (Fig. 6G). In the LD vs. WZS group, 12 lncRNAs were hypermethylated and down-regulated in LD pigs, while 13 lncRNAs were hypermethylated and down-regulated in WZS pigs (Fig. 6H). In addition, we found 29, 47, and 68 lncRNAs that exhibited a positive correlation between DNA methylation and gene expression in the TC vs. LD, TC vs. WZS, and LD vs. WZS groups, respectively. Interestingly, TCONS_00655138, a homolog of human lncRNA TCL1 upstream neural differentiation-associated RNA (*TUNAR*) (Figure S14), was gene body hypermethylated (Fig. 6I) and up-regulated (Fig. 6J) in WZS pigs compared with TC and LD pigs. *TUNAR* was consistently up-regulated in a glucose-dependent manner and was dysregulated in type 2 diabetes^65^; it controls pluripotency and neural differentiation of embryonic stem cells^66^. We proposed that lncRNAs methylation and expression differences might contribute to phenotypic variation of skeletal muscle.

### miRNA-mRNA-methylation interaction networks linked to muscle performance

**Figure 7:**
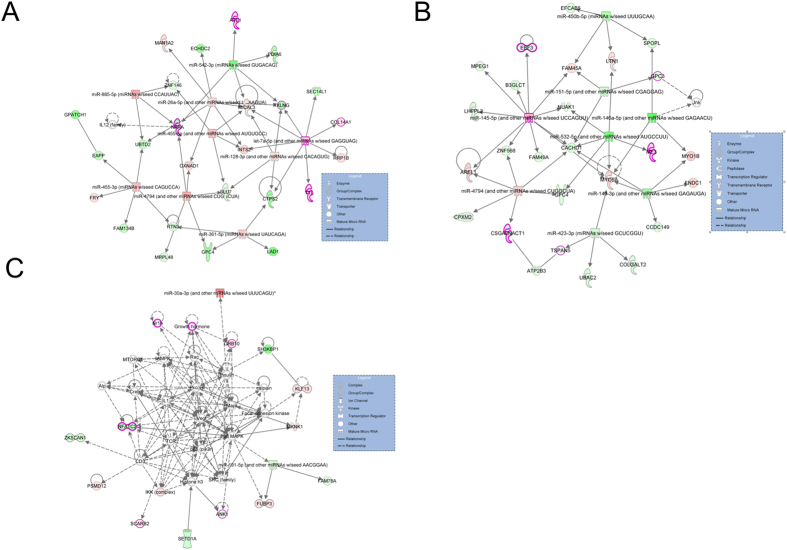
miRNA-mRNA-methylation regulation networks in skeletal muscle among different pig breeds using the IPA software. Gene networks associated with muscle phenotypic variation were identified in TC vs. LD group (**A**), TC vs. LD group (**B**), LD vs. WZS group (**C**), respectively. Graph shows gene symbols with color coding of differentially methylated and expressed genes and mature miRNAs with color coding of differentially expressed miRNAs. The solid lines connecting molecules represent a direct relation and dotted lines an indirect relation. The up-regulated genes or miRNAs are represented in graduation of red, whereas down-regulated genes or miRNAs are shown in graduation of green color based on their fold change in expression level.

In order to understand the consequences of the interaction between methylation, mRNA, and miRNA for phenotypic variation of skeletal muscle, we constructed miRNA-mRNA-methylation interaction networks in skeletal muscle using Ingenuity Pathway Analysis (IPA) software. In the TC vs. LD group, the highest scoring network, based on Fisher’s exact test, had a network score of 73 and was associated with lipid metabolism, and organ morphology; N-acylethanolamine-hydrolyzing acid amidase (*NAAA*), miR-128, and miR-26a were key components in the network (Fig. 7A). Many of the genes in this network are directly or indirectly regulated by each other or miRNAs. For example, *NAAA*, enoyl-CoA hydratase domain containing 2 (*ECHDC2*), and taxilin gamma (*TXLNG*) were down-regulated in LD pigs and negatively regulated by miR-26a. In the TC vs. WZS group, the highest ranking network, scoring 70, was associated with cellular movement, developmental disorder, and endocrine system disorders; this network comprised myosin VA (*MYO5A*), miR-145, miR-143, and myosin IB (*MYO1B*). The predicted targets of miR-143, such as *MYO5A, MYO1B*, and fibronectin type III domain containing 1(*FNDC1*), were up-regulated in WZS pigs compared with TC pigs, while miR-143 was down-regulated (Fig. 7B). In the LD vs. WZS group, the second ranked network, scoring 67, was associated with cellular assembly and organization, cell cycle, DNA replication, recombination, and repair. Moreover, we also found a network, scoring 19, that was associated with cell morphology, organ morphology, and skeletal and muscular system development and function; this network included ankyrin 1 (*ANK1*), growth factor receptor-bound protein 10 (*GRB10*), growth hormone receptor (*GHR*), and miR-191 (Fig. 7C).

### Candidate genes associated with meat, carcass and production traits

GWASs in pigs have reported many candidate SNPs associated with economic traits. Using the animal QTL database^67^, we examined whether candidate genes exhibiting opposite changes in DNA methylation and expression in our study contained SNPs that were significantly associated with meat, carcass, and production traits. The analysis identified 19, 17, and 18 candidate genes associated with meat, carcass, and production traits in the TC vs. LD, TC vs. WZS, and LD vs. WZS group, respectively (Table S15). These genes should be studied further to assess whether they might be considered as candidate biomarkers for pig breeding.

## Discussion

DNA methylation contributes substantially to phenotypic variations in ageing^68^, obesity^69^, and body size^70^. However, the regulatory mechanisms through which DNA methylation influence skeletal muscle performance in mammals remain unclear. In this study, we systematically investigated the genome-wide methylation profile of skeletal muscle from TC (obese-type), LD (lean-type), and WZS (mini-type) pigs by MeDIP-seq. The three selected pig breeds showed significant differences in muscle-related phenotypes. LD pigs exhibit significantly greater muscle mass and lower intramuscular fat content than that of TC pigs, whereas WZS mini pigs have much smaller body size than TC and LD pigs. We performed an integrated analysis of the methylome, mRNAs, lncRNAs, and miRNAs to uncover whether and how DNA methylation changes mediate skeletal muscle phenotypic variations through affecting gene expression.

Though TC pigs are phylogenetically closer to WZS pigs than to LD pigs^71^,^72^, the methylome and transcriptome profiling of skeletal muscle of TC and LD pigs was much more similar to each other than to that of WZS pigs, the reason could possibly (at least in part) be explained by the faster muscle growth rate and larger muscle mass of TC and LD than WZS pigs. We found that the DMGs among TC, LD and WZS pigs were significantly enriched in biological processes and pathways related to lipid metabolism, muscle development, and response to oxidative stress, indicated that differences in DNA methylation among different pig breeds might contribute to muscle phenotypic variations (e.g., muscle mass, fat content, and meat quality). Many genes related to muscle phenotypes were differentially methylated. For example, acetyl-coA carboxylase alpha (*ACACA*) is associated with fatty acid composition, carcass traits, and performance traits in pigs^73^,^74^,^75^. In our study, the *ACACA* gene contained a intron DMR that was hypermethylated in LD pigs compared with TC pigs, as well as another intron DMR that was hypermethylated in WZS pigs compared with LD pigs. Protein kinase, AMP-activated, gamma 2 non-catalytic subunit (*PRKAG2*) regulates cellular energy metabolism and function by inactivating key enzymes involved in regulating biosynthesis of fatty acids and cholesterol. *PRKAG2* was differentially expressed between pigs with divergent phenotypes for fatness traits^76^. The methylation level of intron *PRKAG2* in LD pigs was higher than that of TC pigs. Moreover, significant enrichment of orthologous DMGs related to human height, body mass index, and obesity was observed. These findings should facilitate marker-assisted animal breeding and investigations of human growth, obesity, and muscle-related diseases.

DNA methylation is recognized as a major regulator of gene expression; hypermethylation in promoters and gene body regions can repress gene expression^34^,^35^. We propose that DNA methylation changes might contribute to differences in muscle phenotype by affecting gene expression. We found that genes that exhibited inverse changes in DNA methylation and gene expression in the TC vs. LD group were significantly enriched in GO terms related to oxidation reduction; these differences may, at least in part, underlie the significant differences in lipid metabolism and fat content between TC and LD pigs. DMGs involved in cell proliferation (e.g. *GPC4* and *FGF1*) showed inverse changes in DNA methylation and expression in all three comparisons. Differences in the DNA methylation statuses of these genes may contribute to differences in muscle mass and body size among LC, TD, and WZS pigs. Moreover, several genes known to regulate muscle development and fatty acid metabolism were identified. For example, *FHL1* (four and a half LIM domains 1), an important regulator of skeletal muscle mass^77^, was exon hypermethylated in WZS pigs compared with TC pigs, but exhibited a higher expression level in TC pigs. A previous study revealed that *FHL1* overexpression enhanced myoblast fusion and increased fiber diameter by activating myostatin signaling^78^. Up-regulated expression of *FHL1* in TC pigs may be partially responsible for the relatively great muscle mass of TC pigs in comparison with that of WZS pigs. Histone deacetylase 4 (*HDAC4*) and histone deacetylase 5 (*HDAC5*) are chromatin remodeling enzymes and critical regulators of muscle oxidative and metabolic function; these genes are coordinately down-regulated in metabolic diseases such as obesity^79^. We found that *HDAC4* and *HDAC5* were intron hypomethylated and up-regulated in the LD vs. WZS group, whereas *HDAC4* was intron hypermethylated and down-regulated in the TC vs. LD group. These findings might explain the significant differences in muscle metabolic capacity among TC, LD, and WZS pigs.

LncRNAs are key regulators of skeletal muscle development^56^. For example, linc-MD1, a muscle-specific lncRNA, governs the timing of muscle differentiation^80^. The lncRNA methylation profiles of the pigs in this study were similar to those reported in previous studies, in which exons had higher methylation density than that of introns or promoters^63^,^64^. Functional annotation of neighboring protein coding genes of differentially methylated lncRNAs revealed that the differentially methylated lncRNAs identified in this study were involved in biological processes related to cell apoptosis and cell death, suggesting that DNA methylation changes in lncRNAs might induce functionally relevant changes in skeletal muscle. Moreover, the lncRNAs, which showed significant changes in both DNA methylation and expression, might be considered as candidate genes for future epigenetic studies of skeletal muscle phenotypic variation.

The regulatory networks of methylation, mRNAs, and miRNAs, which determined by both IPA core analysis and target prediction based on evidences in the literature, provided a comprehensive profile of the mechanisms contributing to phenotypic variation of skeletal muscle across different pig breeds. We propose that the DNA methylation and expression changes identified in this study interact with each other and could be functionally relevant for skeletal muscle. The highest scoring network in the TC vs. LD group was associated with lipid metabolism and organ morphology; the *NAAA* gene is central to this network, hypermethylated and down-regulated in LD pigs compared with TC pigs, and negatively regulated by miR-26. *NAAA* plays a key role in the degradation of N-acylethanolamines, which are ethanolamides of long-chain fatty acids^81^. miR-26 expression has been reported to promote energy dissipation and differentiation of myoblasts^82^,^83^. Moreover, this network suggests that higher energy dissipation and muscle differentiation ability in skeletal muscle of LD pigs may result in less intramuscular fat deposition and more muscle mass. In the TC vs. WZS group, the top gene network was associated with cellular movement and developmental disorder. miR-143–3p, which was down-regulated in WZS pigs compared with TC pigs, has been reported to regulate muscle fiber differentiation in skeletal muscle^82^ and targeted *MYO5A* and *MYO1B* in the top network in the TC vs. WZS group. *MYO5A* and *MYO1B* are members of the myosin gene superfamily, which is critical for muscle contraction and cell movement^84^. These facts may suggest the potential regulatory pattern for the differences in muscle fiber characteristics between TC and WZS pigs. In addition, the network involving skeletal and muscular system development and function in the LD vs. WZS group highlighted the importance of p38 MAPK signaling in the regulation of phenotypic variation in muscle mass and body size between LD and WZS pigs. The p38 MAPK signaling pathway is a major regulator of skeletal muscle development^85^. *GRB10* and *ANK1*, components of the p38 MAPK signaling pathway, were included in the network. *GRB10* regulates fiber number during skeletal muscle development^86^ and its deletion could promote muscle cell proliferation and differentiation^87^. Greater expression of *GRB10* as a result of intron hypomethylation in WZS pigs might be a reason this breed has reduced muscle mass in comparison with that of LD pigs. *ANK1* was differentially expressed in prenatal porcine muscle and has been identified as a candidate gene associated with meat quality and muscle deposition^88^. In this study, *ANK1* was intron hypermethylated and down-regulated in WZS pigs compared with TC and LD pigs, implying that hypermethylation of *ANK1* and reduced *ANK1* expression in WZS pigs partially underlies the reduced body size of WZS pigs in comparison with that of TC and LD pigs. The results of integrative network analysis should facilitate systematic exploration of the roles of DNA methylation and miRNAs in muscle phenotypic variation among pig breeds.

In this study, we systematically integrated genome-wide DNA methylation and expression profiles of mRNAs, lncRNAs, and miRNAs in the skeletal muscle of three pig breeds with different phenotypes, illuminating the potential mechanisms underlying muscle phenotypic variation and identifying new candidate genes associated with skeletal muscle performance. Our study provides a resource of mRNAs and non-coding RNAs that can be used for animal breeding and biomedical research related to growth, obesity, and muscle-related diseases. However, pooling samples were used for library construction in our study, further studies are still needed to evaluate the effect of methylation and gene expression difference on skeletal muscle within breeds.

## Materials and Methods

### Sample collection and DNA preparation

All animal procedures were conducted according to guidelines approved by HuBei Province, P.R. China for the Biological Studies Animal Care and Use Committee. Three adult female pigs (after birth 240 days) of each breed (TC, LD, and WZS) were humanly slaughtered at a commercial slaughterhouse. Unrelated individuals of each breed were chosen based on their pedigrees. All pigs were raised under the same feeding and management practices at the same farm. The *longissimus dorsi* muscle samples were collected, frozen in liquid nitrogen, and stored at −80 °C. Genomic DNA was isolated by phenol-chloroform extraction. The quality and concentration of DNA were evaluated by agarose gel electrophoresis and spectrophotometry.

### Methylated DNA immunoprecipitation sequencing

TC, LD, and WZS libraries were constructed by mixing equal amounts DNA from three individuals within each breed following a previously described protocol. Genomic DNA was sonicated to generate fragments of approximately 100–500 bp using a Covarias sonication system. Libraries were constructed using a Paired-End DNA Sample Prep kit (Illumina, CA, USA) according to the manufacturer’s instructions. Adaptor-ligated DNA was used for subsequent MeDIP enrichment with an antibody that specifically recognizes 5′-methylcytosine. The enriched methylated fragments were purified on DNA Clean & Concentrator-5 columns (Zymo Research, Orange, CA, USA). DNA from qualifying MeDIP experiments was amplified by adaptor-mediated PCR to produce libraries with insert sizes between 220 and 300 bp. Amplification quality and quantity were evaluated using an Agilent 2100 Analyzer and the DNA 1000 Nano Chip Kit (Agilent Technologies, Santa Clara, CA, USA). Each MeDIP library was subjected to paired-end sequencing with 49-bp reads on an Illumina Hiseq 2000 Sequencing System (Illumina, San Diego, CA, USA) following the manufacturer’s instructions (BGI, Shenzhen, China).

### Sequencing data analysis

The raw data were aligned to *Sus scrofa* genome assembly 10.2 with bowtie2 (version 2.1.0) with default parameters^89^. The Picard toolkit was applied to remove PCR duplicates (version 1.118; http://broadinstitute.github.io/picard/). The ngs.plot program was used to visualize the read coverage of MEDIP around each gene body and TSS region^90^. MeDIP quality assessment and DNA methylation score (Reads Per Kilobases per Million reads (RPKM) value) calculation were performed using the Bioconductor package MEDIPS at 250-bp windows after extending each read to a length of 300 bp along the sequencing direction^45^. The resulting coverage profiles were exported as wiggle files. Differentially methylated regions (DMRs) were identified with the criteria of FDR adjusted P < 0.05 by edgeR (exact test for negative binomial distribution) integrated in MeDIPs. Of note, in our study, *hypermethylated* indicated that the methylation level of the second group in the comparison was higher than that of the first group, whereas *hypomethylated* indicated that the methylation level of the first group in the comparison was higher than that of the second group. The annotation of DMRs were performed by comparing the chromosome coordinate of DMRs with the corresponding annotation information using the intersect tool from the BEDTools suite^91^. Gene annotation information was downloaded from the Ensembl database (version 78). Annotation information for CpGi in the pig genome was downloaded from the UCSC public FTP site. In this study, we defined the genomic region from −2000 to the TSS as the promoter region and from the TSS to TTS as the gene body region. Venn diagrams were generated using Venny 2.1.0 (http://bioinfogp.cnb.csic.es/tools/venny/index.html) to visualize overlapping gene sets.

### Bisulfite sequencing polymerase chain reaction

Genomic DNA was treated with sodium bisulfite using the EZ DNA Methylation-GoldTM Kit (Zymo Research, Irvine, CA, USA) according to the manufacturer’s protocol. BSP primers were designed using the MethPrimer program (http://www.urogene.org/methprimer/)^92^ and listed in Table S16. PCR amplification of selected regions was performed in a 20-mL reaction volume containing 2 μL modified DNA, 7 μL RNase-free water, 0.5 μL of both forward and reverse primers, and 10 μL HotStarTaq Master Mix (Qiagen, USA). The PCR reactions were run under the following cycling conditions: initial denaturation at 95 °C for 5 min, followed by 35 cycles of denaturation at 94 °C for 30 s, annealing at 55 °C for 60 s, and elongation at 72 °C for 60 s. The PCR products were gel-purified using a Gel Extraction Kit (Tiangen, Beijing, China) and subcloned into the pMD18-T vector (Takara, Osaka, Japan). Seven to twelve clones of each sample were randomly selected for DNA sequencing. Bisulfite sequencing data were analyzed and visualized using BIQ Analyzer software^93^.

### RNA-seq and miRNA-seq datasets

mRNA and miRNA profiles from the skeletal muscle of adult TC, LD, and WZS pigs were obtained from our previous study^46^. The raw data are available from the NCBI SRA database under accession no. SRP058340. Same samples were used for the MeDIP-seq, RNA-seq, and miRNA-seq analysis. When calculating the correlation between gene expression and methylation, we only considered genes with expression differences of at least 1.5-fold. For the global methylation and expression correlation analysis, we divided the genes into five groups according their expression level: 0 (silent genes), 0–1 (lowly expressed genes), 1–10 (moderately expressed genes), 10–100 (highly expressed genes), and >100 (highest expressed genes).

### LncRNA expression analysis

LncRNAs were identified and annotated using our RNA-seq analysis pipeline across 9 organs and 3 developmental skeletal muscle, the RNA-seq data are available at the NCBI Gene Expression Omnibus under accession number GSE73763. Briefly, the consensus transcriptome of pigs was reconstructed using TopHat mapping^94^ followed by Cufflinks assembly^95^. And then the annotated, short, single-exon and unreliable transcripts, as well as those having the potential to encode proteins or homology with canonical structural RNAs, were removed. We used htseq-count^96^ to count lncRNAs reads, after which expression levels were calculated by the RPKM method. The annotation and expression of lncRNAs in the three pig breeds were shown in Table S11. LncRNAs with fold-change ≥2 and FDR < 0.05 were considered as differentially expressed. The FDR values were adjusted by the Benjamini-Hochberg method. LncRNAs with expression differences of at least 1.5-fold were selected for integrative analysis with DNA methylation. The genes neighboring each lncRNA were identified as the nearest protein coding genes upstream and downstream of the lncRNA loci (within < 10 kb).

### Gene ontology and KEGG pathway enrichment analysis

Gene ontology (GO) and KEGG pathway enrichment analyses were performed using Bioconductor package GOstats^97^. *P*-values were corrected for multiple hypothesis testing using Benjamini–Hochberg algorithm by p.adjust function in R. The FDR-corrected *P*-value below 0.05 was considered to be statistically significant. Ensembl gene IDs were converted into human gene symbols with Biomart (http://www.biomart.org/) before performing the enrichment analyses because the pig genome database was poorly annotated.

### Interaction network construction

Ingenuity Pathways Analysis (IPA) software (Qiagen, USA) was used to construct miRNA-methylation-mRNA interaction networks based on evidences in the literature to identify the extent of gene connectivity in skeletal muscle with a cutoff of 35 molecules per network and 25 networks per analysis. For most of the muscle development or lipid metabolism related DMGs exhibited inverse changes between methylation and expression, the networks were constructed using the following criteria: 1) miRNAs were differentially expressed between different pig breeds; 2) mRNAs showed significant inverse differences in methylation and gene expression; 3) targets of differentially expressed miRNAs between different pig breeds were predicted by the “target filter” tool in the IPA software application, which relies on three popular algorithms (TargetScan, TarBase, and miRecords). The network score is based on the hypergeometric distribution and is calculated with the right-tailed Fisher’s Exact Test. The score is the −log of Fisher’s Exact test P value, with the higher the score corresponding to a lower the probability of finding the observed number of network eligible molecules in a given network by random chance.

### Accession codes

The MeDIP-seq raw data from this study have been deposited in NCBI Sequence Read Archive with accession number SRP080038 (http://www.ncbi.nlm.nih.gov/Traces/sra/).

## Additional Information

**How to cite this article**: Yang, Y. *et al*. Comparative analysis of DNA methylome and transcriptome of skeletal muscle in lean-, obese-, and mini-type pigs. *Sci. Rep.* **7**, 39883; doi: 10.1038/srep39883 (2017).

**Publisher's note:** Springer Nature remains neutral with regard to jurisdictional claims in published maps and institutional affiliations.

## Supplementary Material

Supplementary Materials

Supplementary Tables
