# Supplementary material for: Comparative analysis of DNA methylome and transcriptome of skeletal muscle in lean-, obese-, and mini-type pigs
Source: Sci Rep. 2017 Jan 3;7:39883. doi: 10.1038/srep39883 (PMC5206674; doi:10.1038/srep39883)
Supplement: Supplementary Materials [file srep39883-s1.doc]

**Supplementary Information for**

Comparative analysis of DNA methylome and transcriptome of skeletal muscle in lean-, obese-, and mini-type pigs

Yalan Yang^1,2^, Guoming Liang^1,2^, Guanglin Niu^1,2^, Yuanyuan Zhang^1,2^, Rong Zhou^1^, Yanfang Wang^1^, Yulian Mu^1^, Zhonglin Tang^1,2^*, Kui Li^1,2^*

1. State Key Laboratory of Animal Nutrition, Institute of Animal Science, Chinese Academy of Agricultural Sciences, Beijing 100193, China.

2. Agricultural Genome Institute at Shenzhen, Chinese Academy of Agricultural Sciences, Shenzhen, 518124, China.

*Corresponding author: Zhonglin Tang, zhonglinqy_99@sina.com; Kui Li, likui@caas.cn

**Supporting information**

**Figure S1.** High-quality 49bp paired-end MeDIP-seq data was obtained for all samples

**Figure S2.** Saturation analysis of MeDIP-seq data.

**Figure S3.** Coverage analysis of MeDIP-seq data.

**Figure S4.** Correlation analysis of the methylome in different elements across breeds

**Figure S5.** Validation of a high methylated region in the *RHBDF1* gene on Chromosome 3 from 40,775,833-40,776,259 by bisulfite sequencing PCR.

**Figure S6.** Validation of a moderate methylated region in the *COL5A1* gene on Chromosome 2 from 69,163,025-69,163,484 by bisulfite sequencing PCR.

**Figure S7.** Validation of a differentially methylated region on Chromosome 17 from 58,818,233-58,818,614 by bisulfite sequencing PCR.

**Figure S8.** Validation of a differentially methylated region on Chromosome 16 from 13,816,664-13,816,844 by bisulfite sequencing PCR.

**Figure S9.** Validation of a differentially methylated region on Chromosome 3 from 91,159,897-91,160,240 by bisulfite sequencing PCR.

**Figure S10.** Validation of a differentially methylated region on Chromosome 3 from 40,869,424-40,870,021 by bisulfite sequencing PCR.

**Figure S11.** GO analysis of shared DMGs in all three comparisons.

**Figure S12.** DNA methylation level distributions in the genebody region of five levels of gene expression.

**Figure S13.** GO analysis of protein-coding genes that are neighbors of differentially methylated lncRNAs.

**Figure S14.** Alignment results of TCONS_00655138 with human *TUNAR* gene.

**Table S1**. Differentially methylated regions between Tongcheng and Landrace pigs.

**Table S2**. Differentially methylated regions between Tongcheng and Wuzhishan pigs.

**Table S3**. Differentially methylated regions between Landrace and Wuzhishan pigs.

**Table S4**. Gene ontology analysis of differentially methylated genes between Tongcheng and Landrace pigs.

**Table S5**. Gene ontology analysis of differentially methylated genes between Tongcheng and Wuzhishan pigs.

**Table S6**. Gene ontology analysis of differentially methylated genes between Landrace and Wuzhishan pigs.

**Table S7**. Differentially methylated genes associated with human body size, obesity and related traits. The methylation level of these genes could be seen in the Table S1-S3.

**Table S8**. Differentially methylated lncRNAs between Tongcheng and Landrace pigs.

**Table S9**. Differentially methylated lncRNAs between Tongcheng and Wuzhishan pigs.

**Table S10**. Differentially methylated lncRNAs between Landrace and Wuzhishan pigs.

**Table S11**. LncRNA expression profile of Tongcheng, Landrace and Wuzhishan pigs.

**Table S12**. Differentially expressed lncRNAs between Tongcheng and Landrace pigs.

**Table S13**. Differentially expressed lncRNAs between Tongcheng and Wuzhishan pigs.

**Table S14**. Differentially expressed lncRNAs between Landrace and Wuzhishan pigs.

**Table S15.** Candidate genes associated with meat, carcass and production traits

.


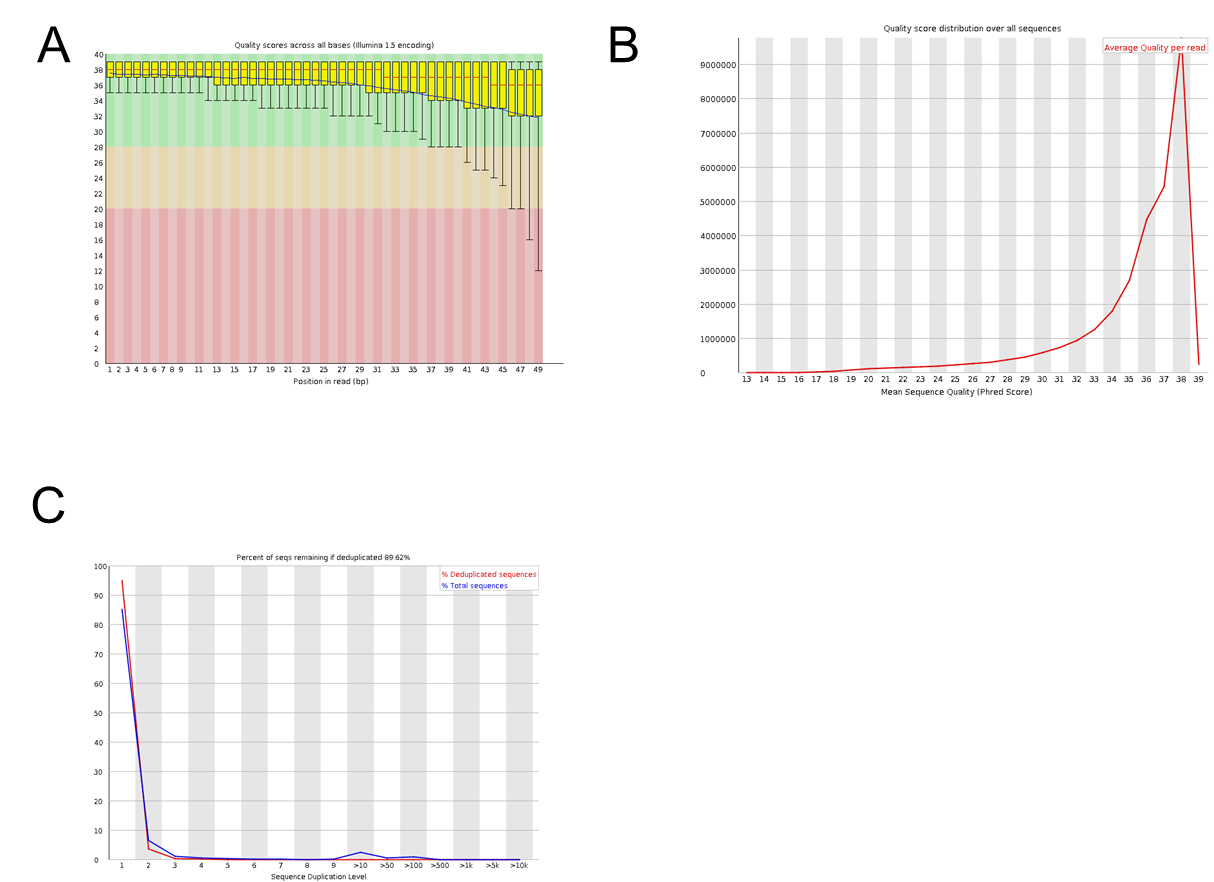


**Figure S1.** High-quality 49bp paired-end MeDIP-seq data was obtained for all samples. (A) A representative example of per base sequence quality, the central red line is the median value, the yellow box represents the inter-quartile range (25-75%). and the upper and lower whiskers represent the 10% and 90% points while the blue line represents the mean quality; (B) A representative example of average sequence quality (demonstrating that the overwhelming majority of sequences have high quality values), (C) A representative example of read duplication (demonstrating a low frequency of clonally-duplicated reads).

**
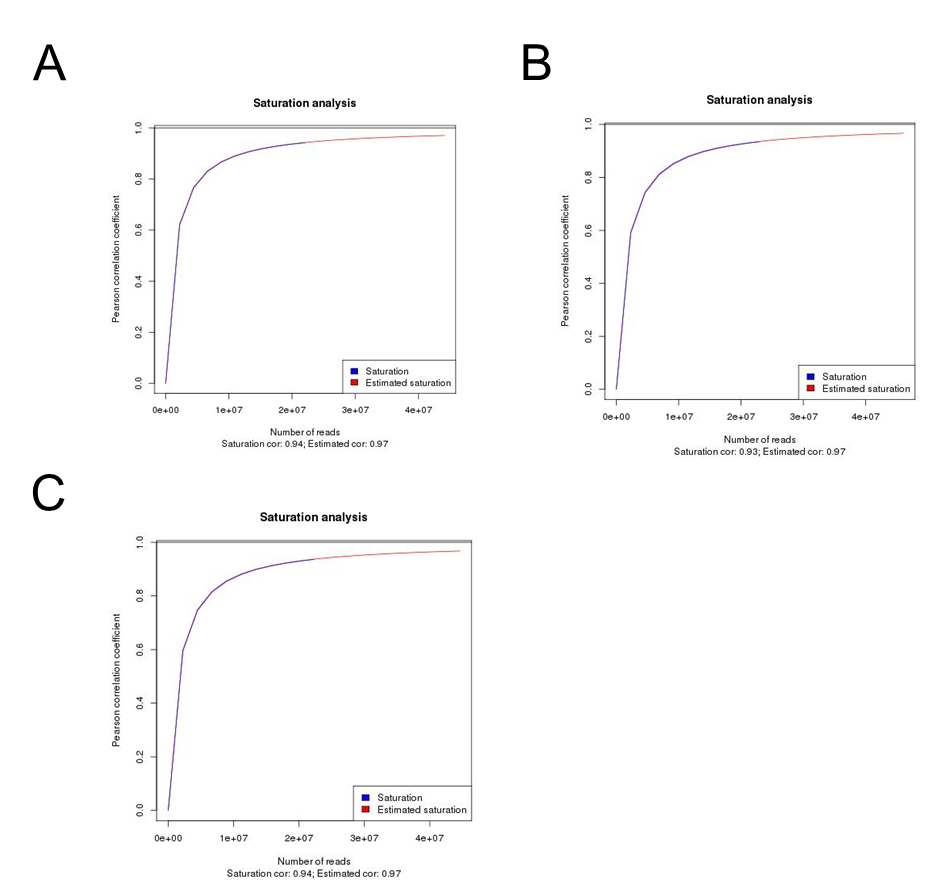
**

**Figure S2.** Saturation analysis of MeDIP-seq data. (A) Tongcheng pigs. (B) Landrace pigs. (C) Wuzhishan pigs.


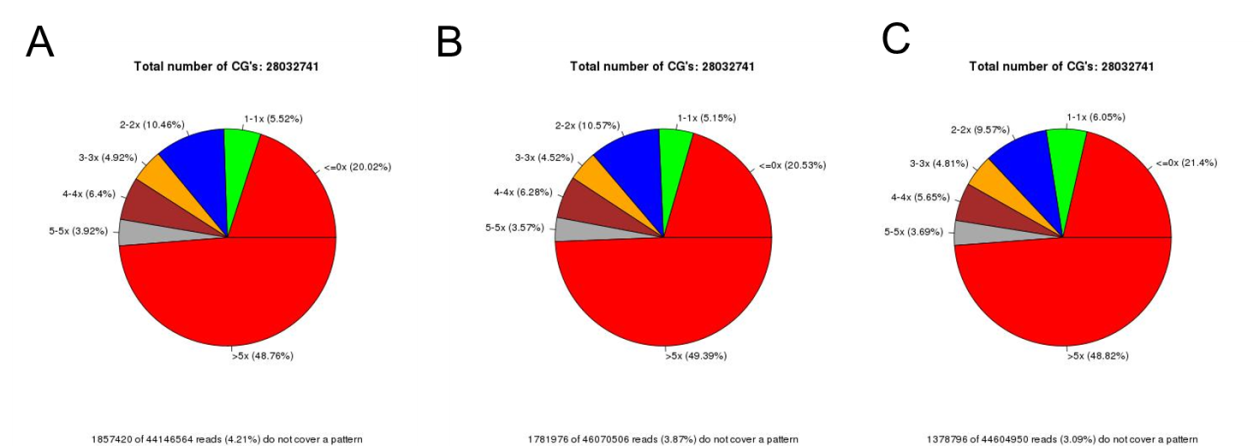


**Figure S3.** Coverage analysis of MeDIP-seq data. (A) Tongcheng pigs. (B) Landrace pigs. (C) Wuzhishan pigs.


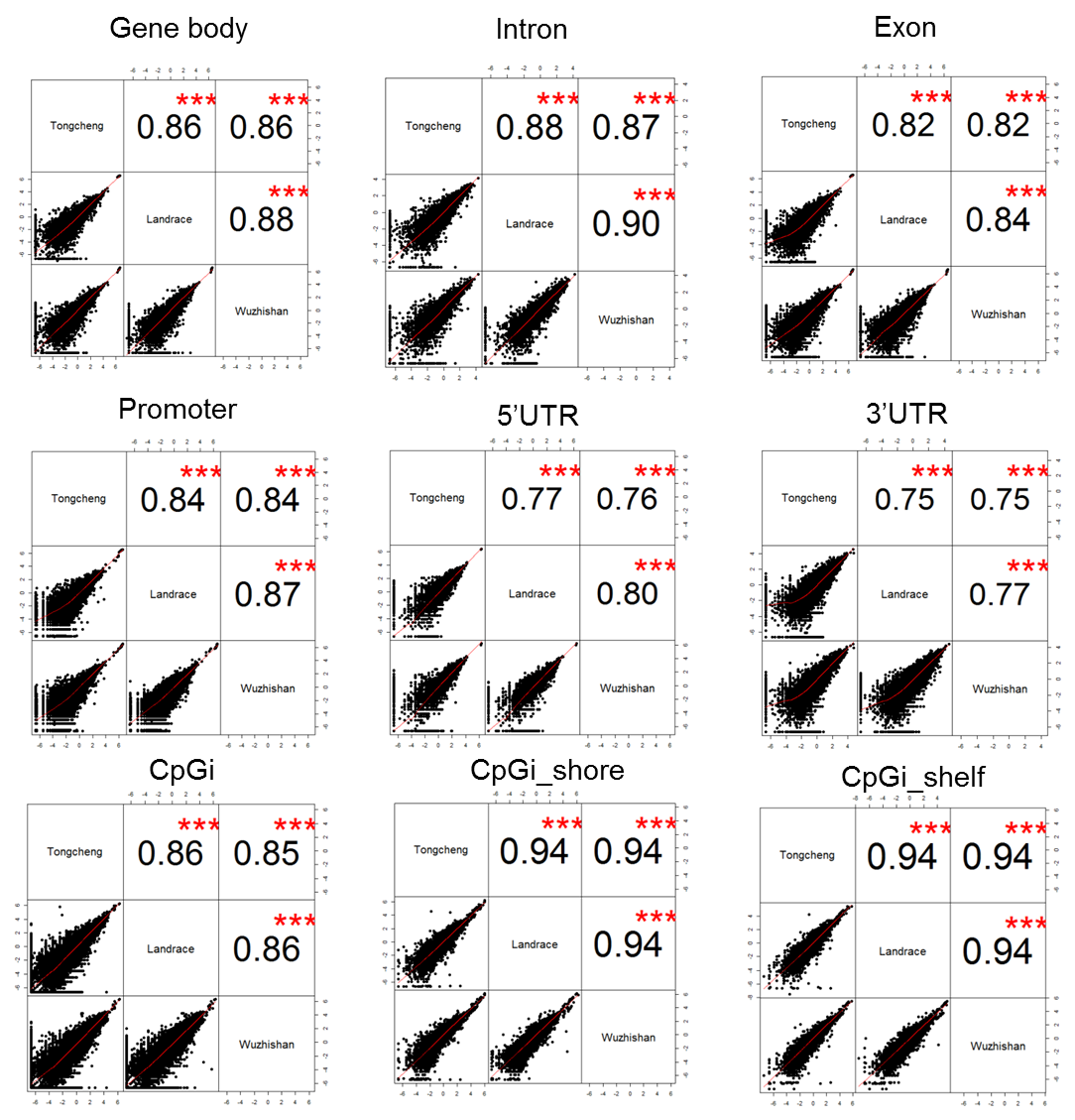


**Figure S4.** Correlation analysis of the methylome in different elements across breeds


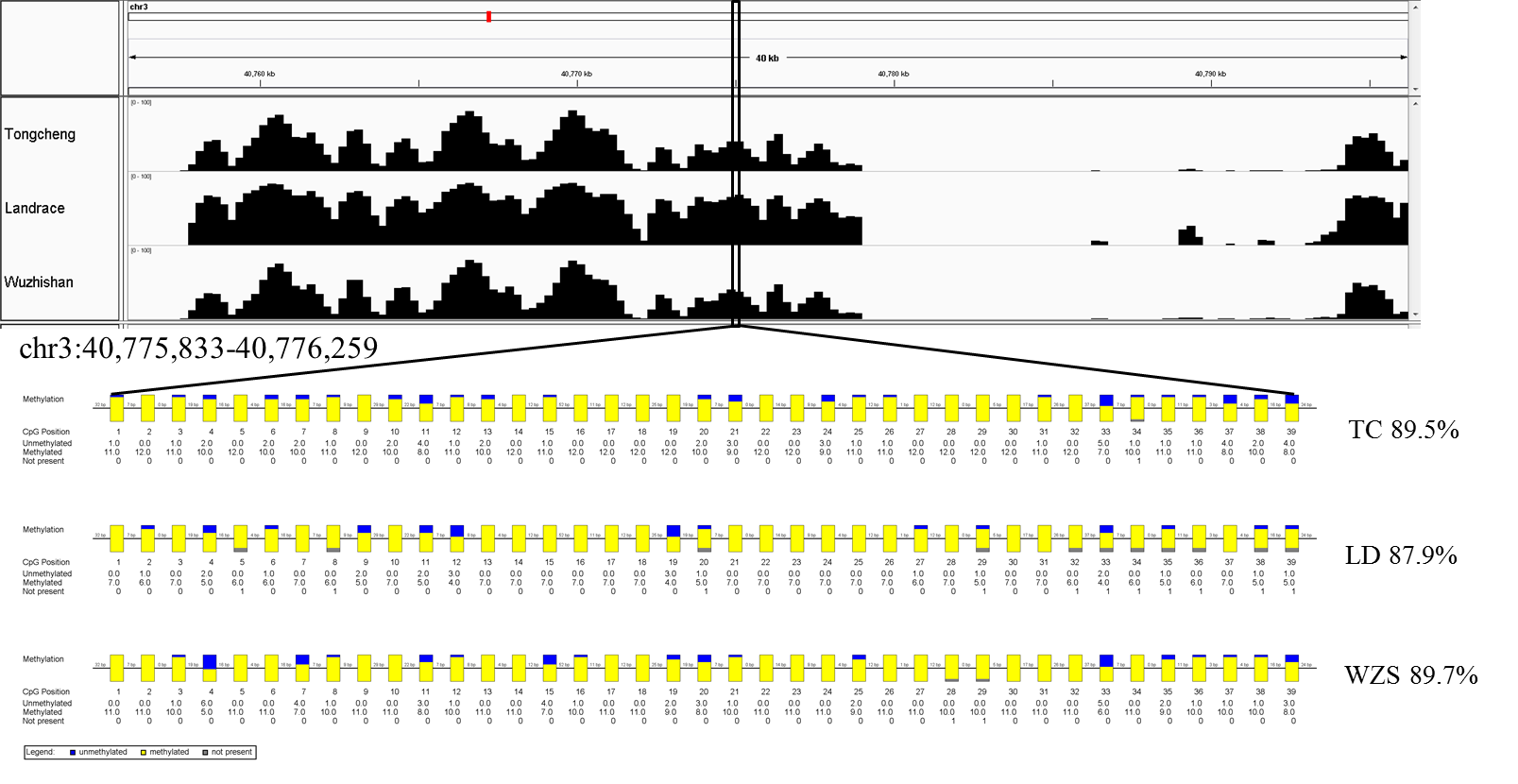


**Figure S5.** Validation of a high methylated region on Chromosome 3 from 40,775,833-40,776,259 by bisulfite sequencing PCR.


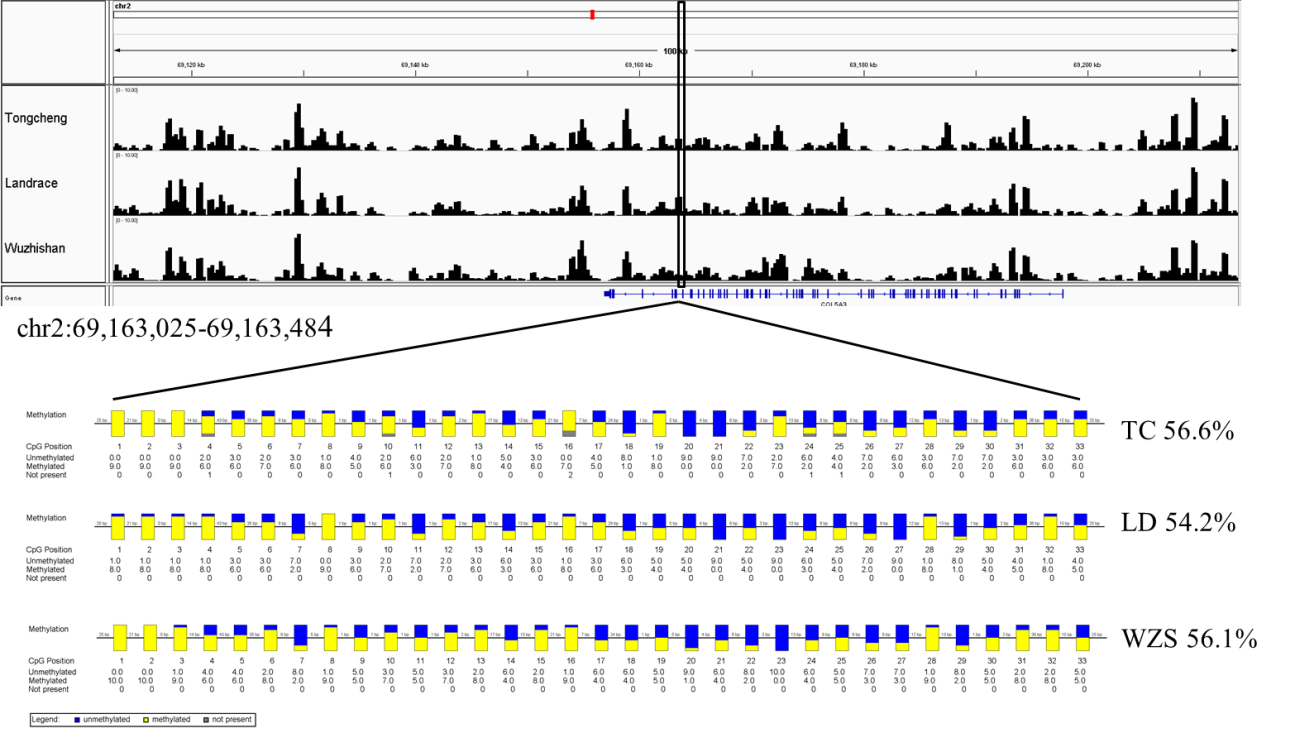


**Figure S6.** Validation of a moderate methylated region on Chromosome 2 from 69,163,025-69,163,484 by bisulfite sequencing PCR.


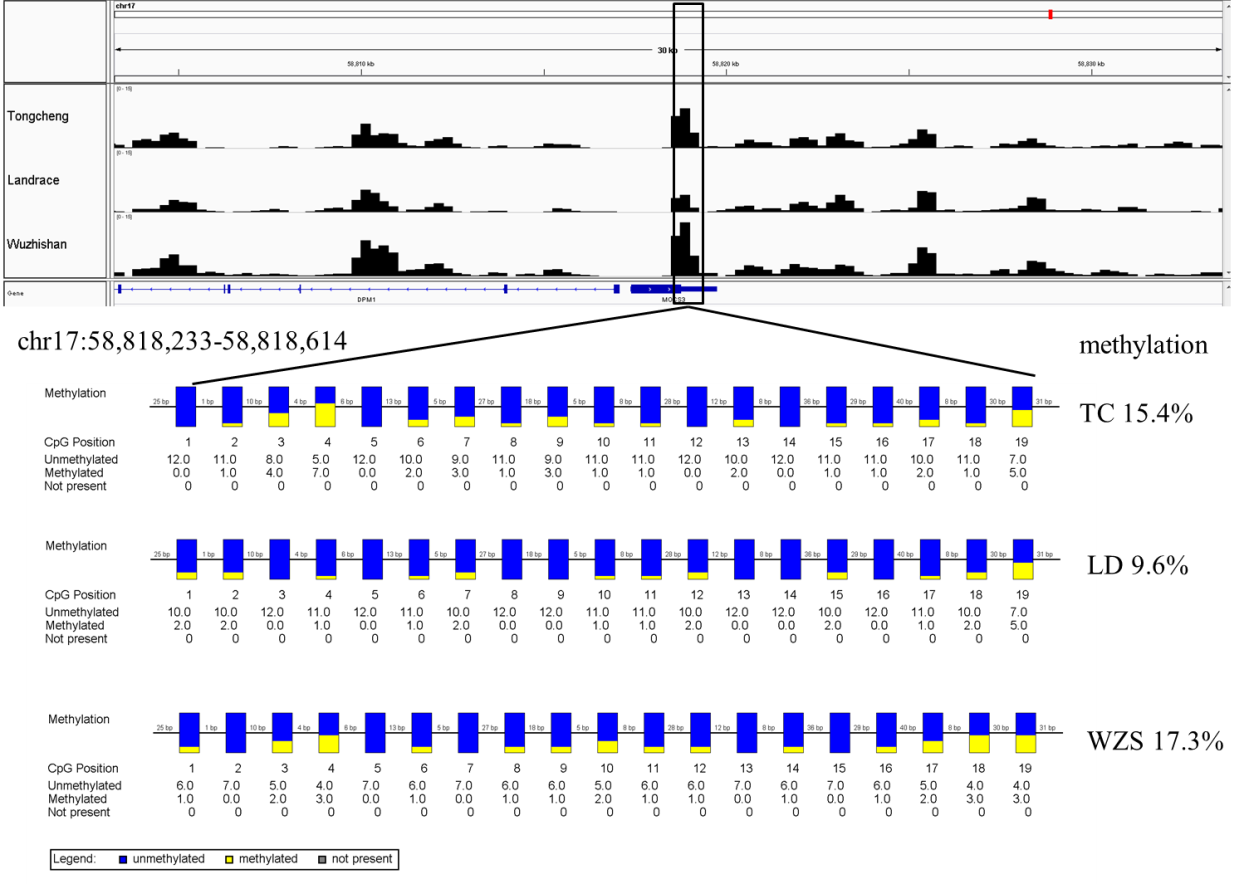


**Figure S7.** Validation of a differentially methylated region on Chromosome 17 from 58,818,233-58,818,614 by bisulfite sequencing PCR.


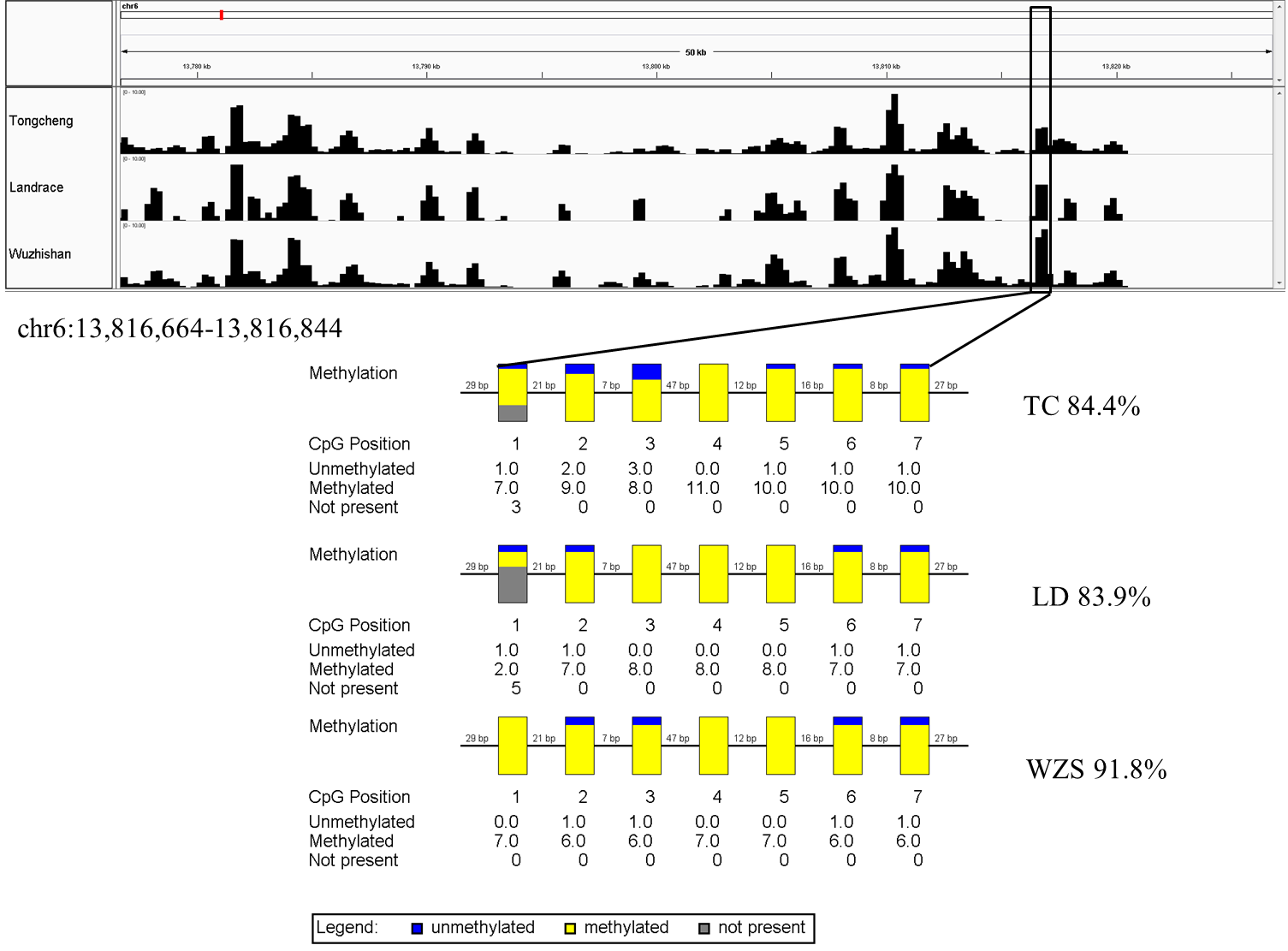


**Figure S8.** Validation of a differentially methylated region on Chromosome 16 from 13,816,664-13,816,844 by bisulfite sequencing PCR.


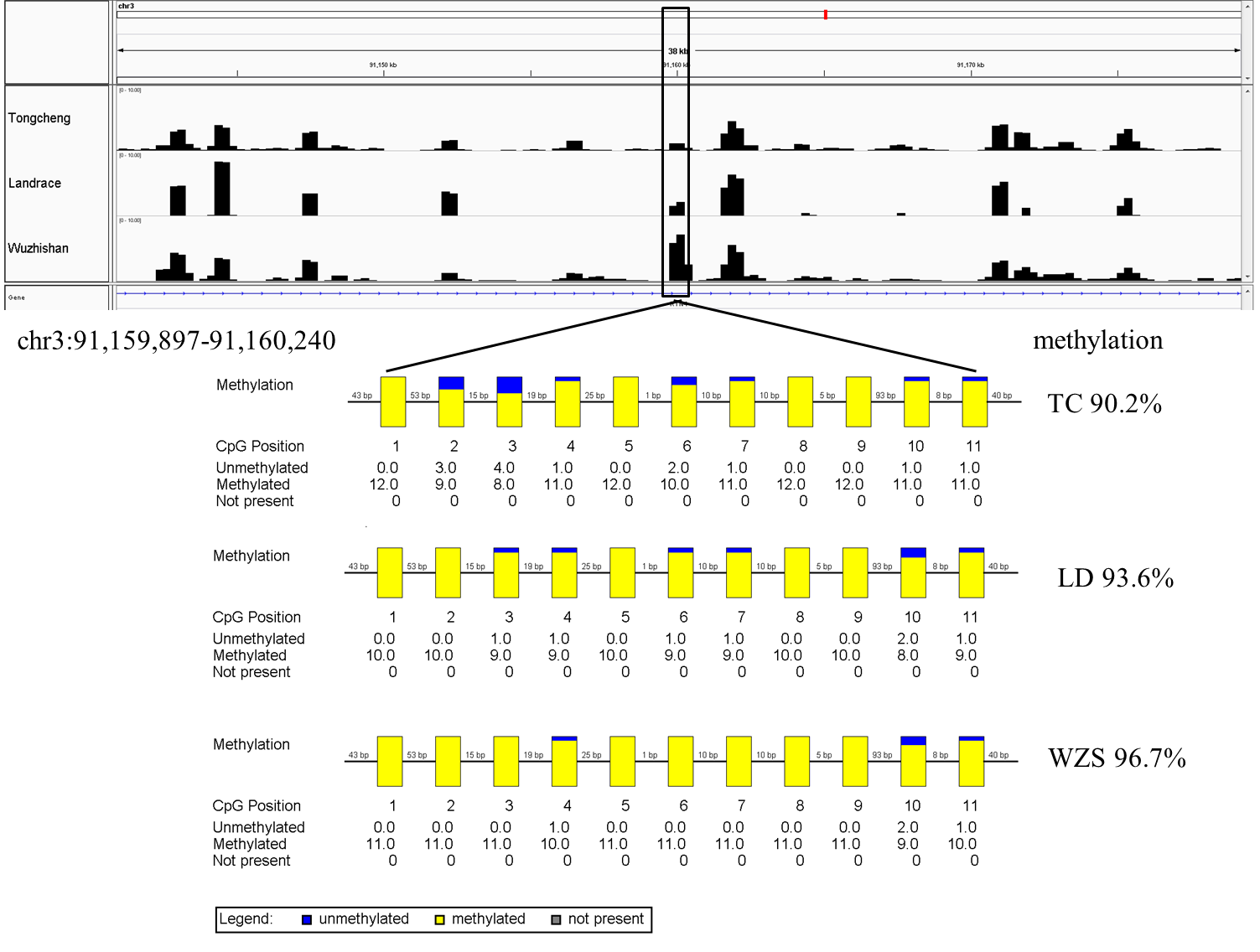


**Figure S9.** Validation of a differentially methylated region on Chromosome 3 from 91,159,897-91,160,240 by bisulfite sequencing PCR.


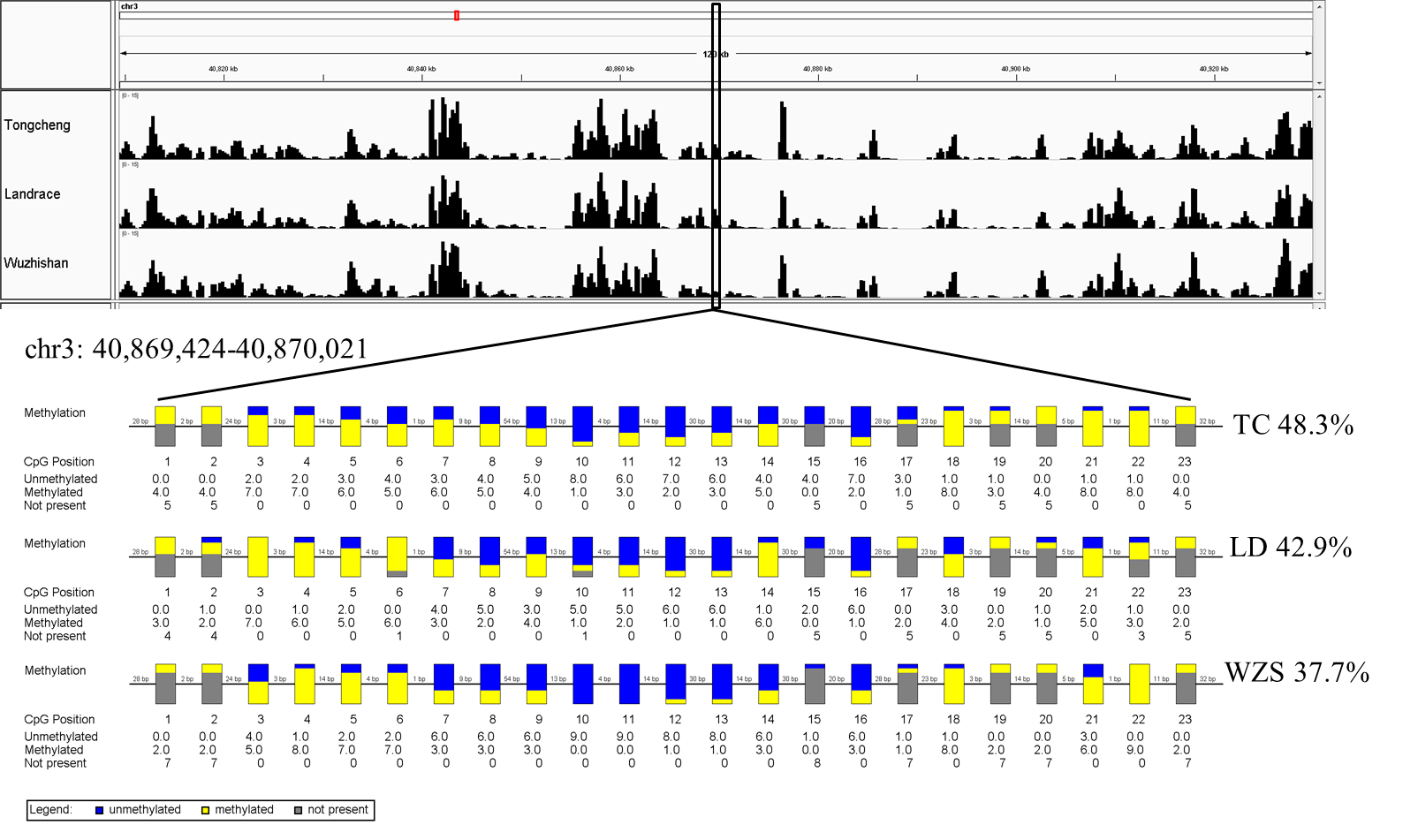


**Figure S10.** Validation of a differentially methylated region on Chromosome 3 from 40,869,424-40,870,021 by bisulfite sequencing PCR.


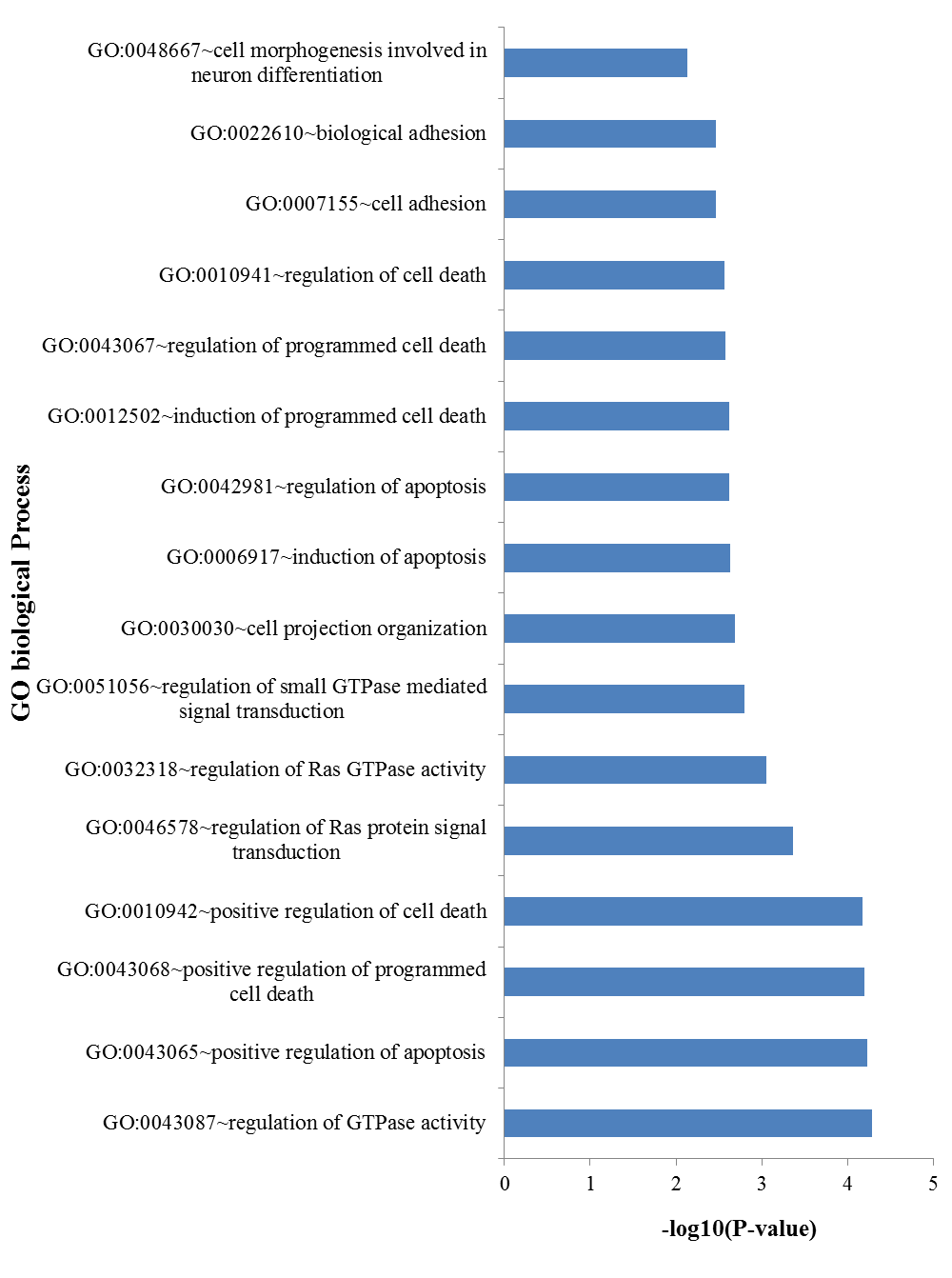


**Figure S11.** GO analysis of shared DMGs in all three comparisons.

A


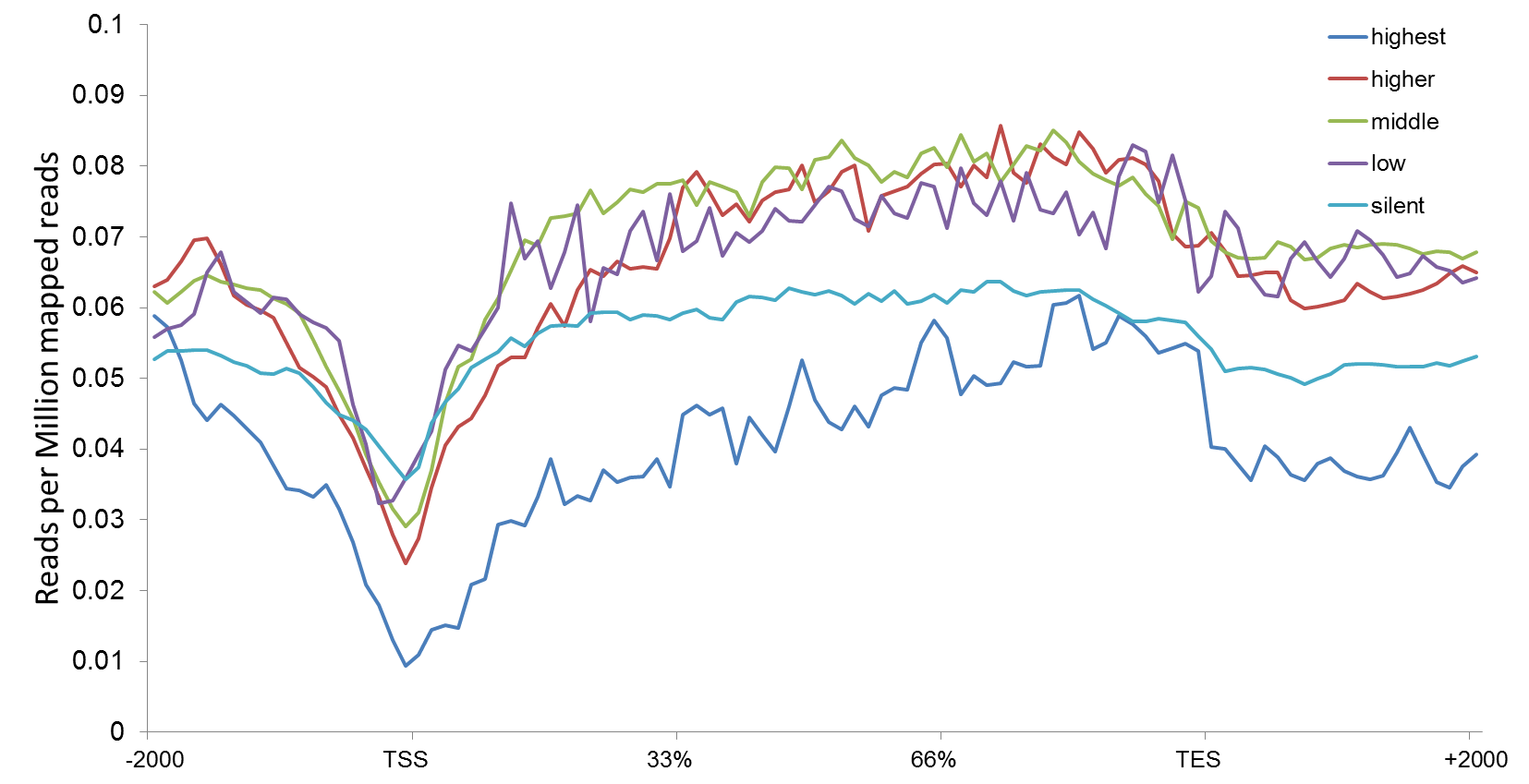


B


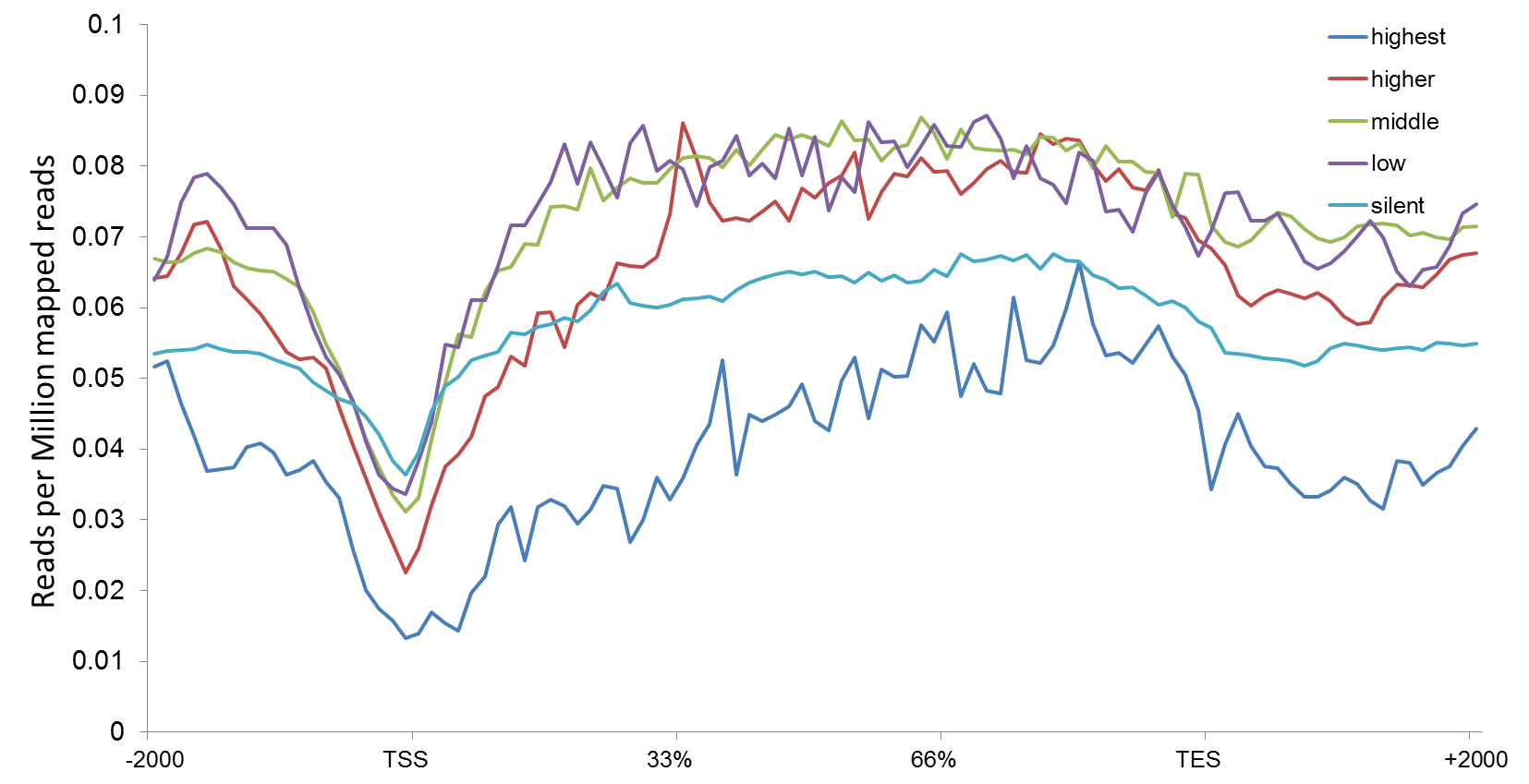


C


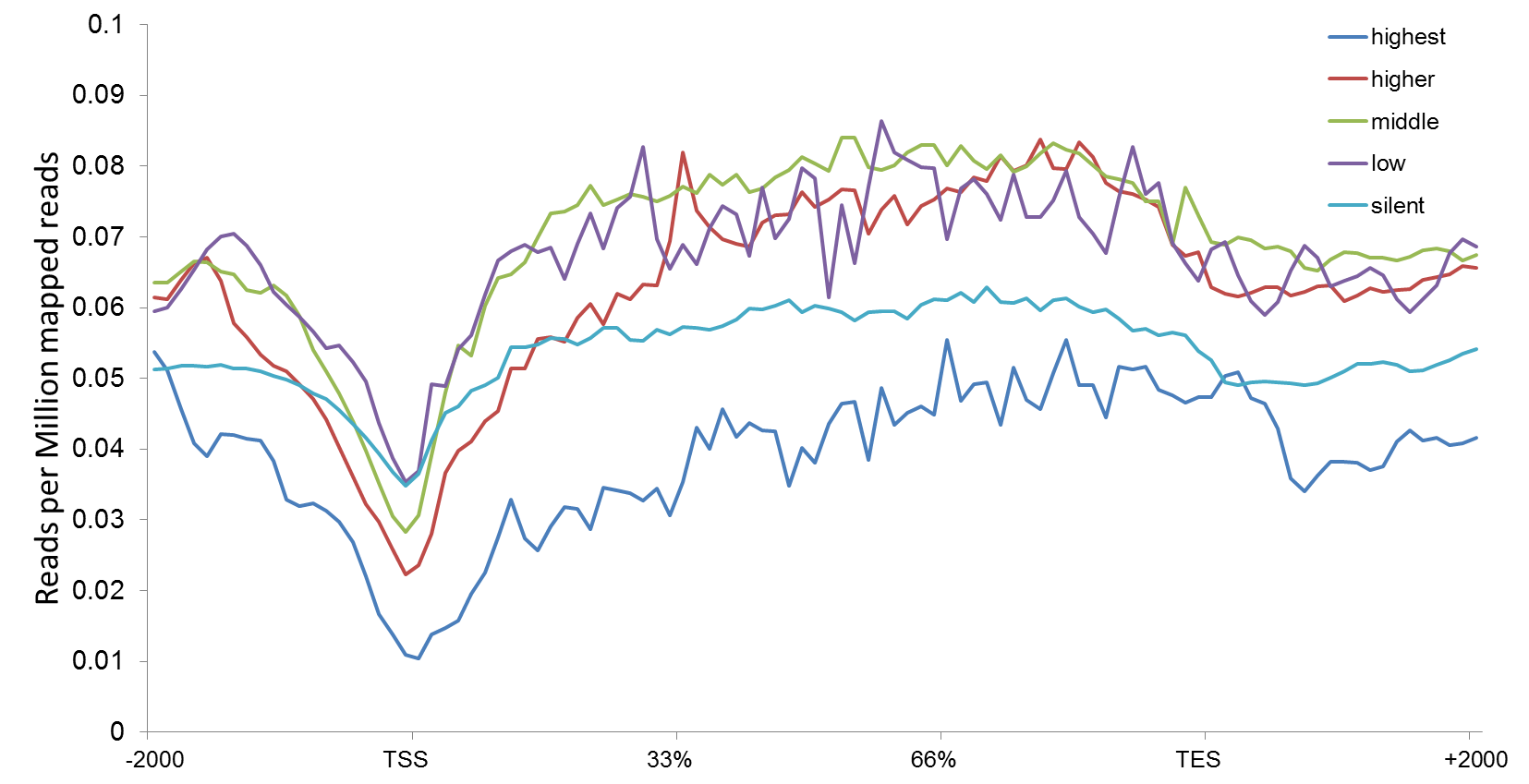


**Figure S12.** DNA methylation level distributions in the genebody region of five levels of gene expression. (A): Tongcheng pig; (B): Landrace pig, (C) : Wuzhishan pig. In upstream and downstream 2 kb regions, the regions were split into 20 non-overlapping windows, In the gene body, each gene was split into 40 equal windows, and the reads per million mapped reads was calculated for each window.


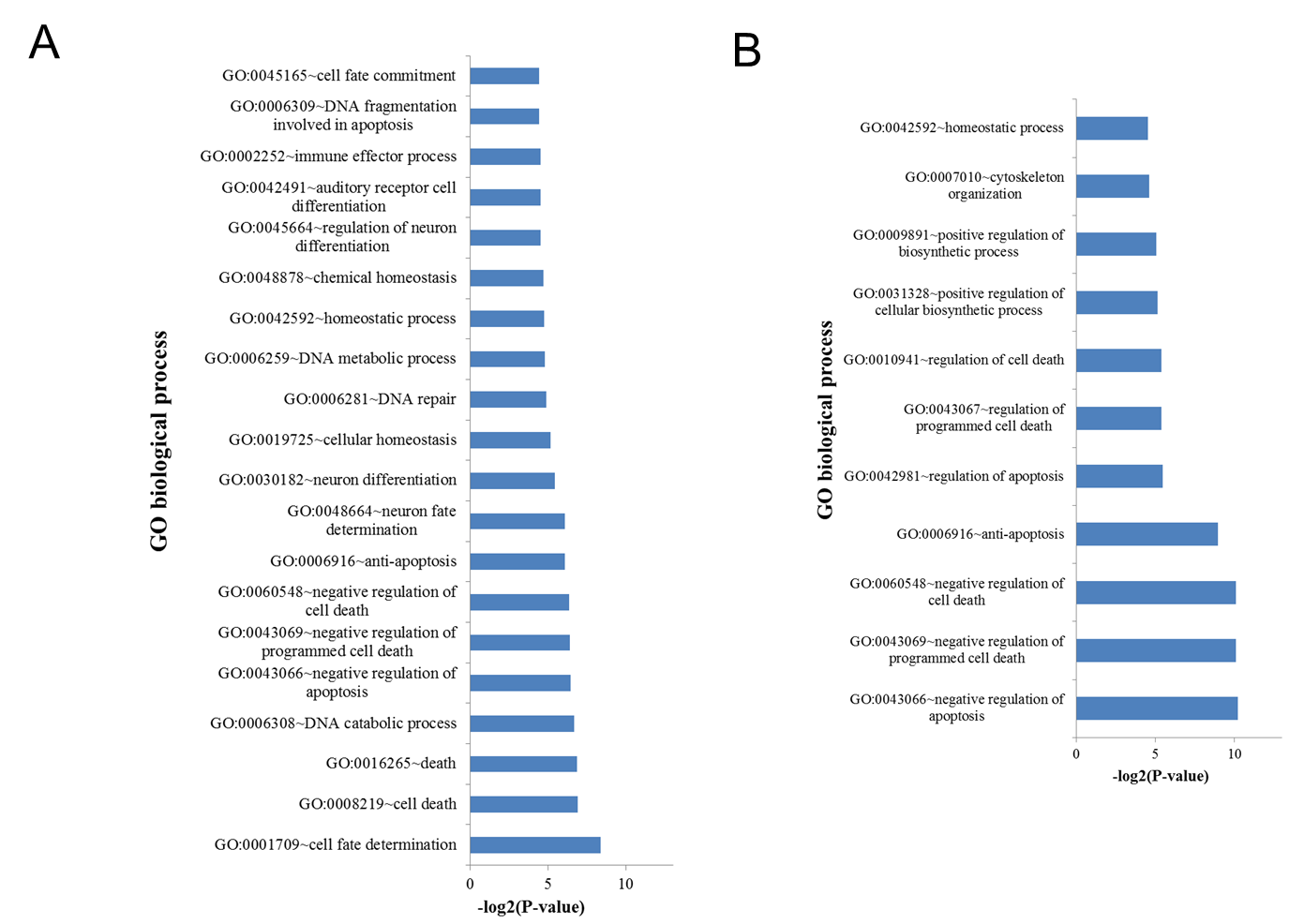


**Figure S13.** GO analysis of protein-coding genes that are neighbors of differentially methylated lncRNAs in TC vs. WZS pigs **(A)**, in LD vs. WZS pigs **(B)**


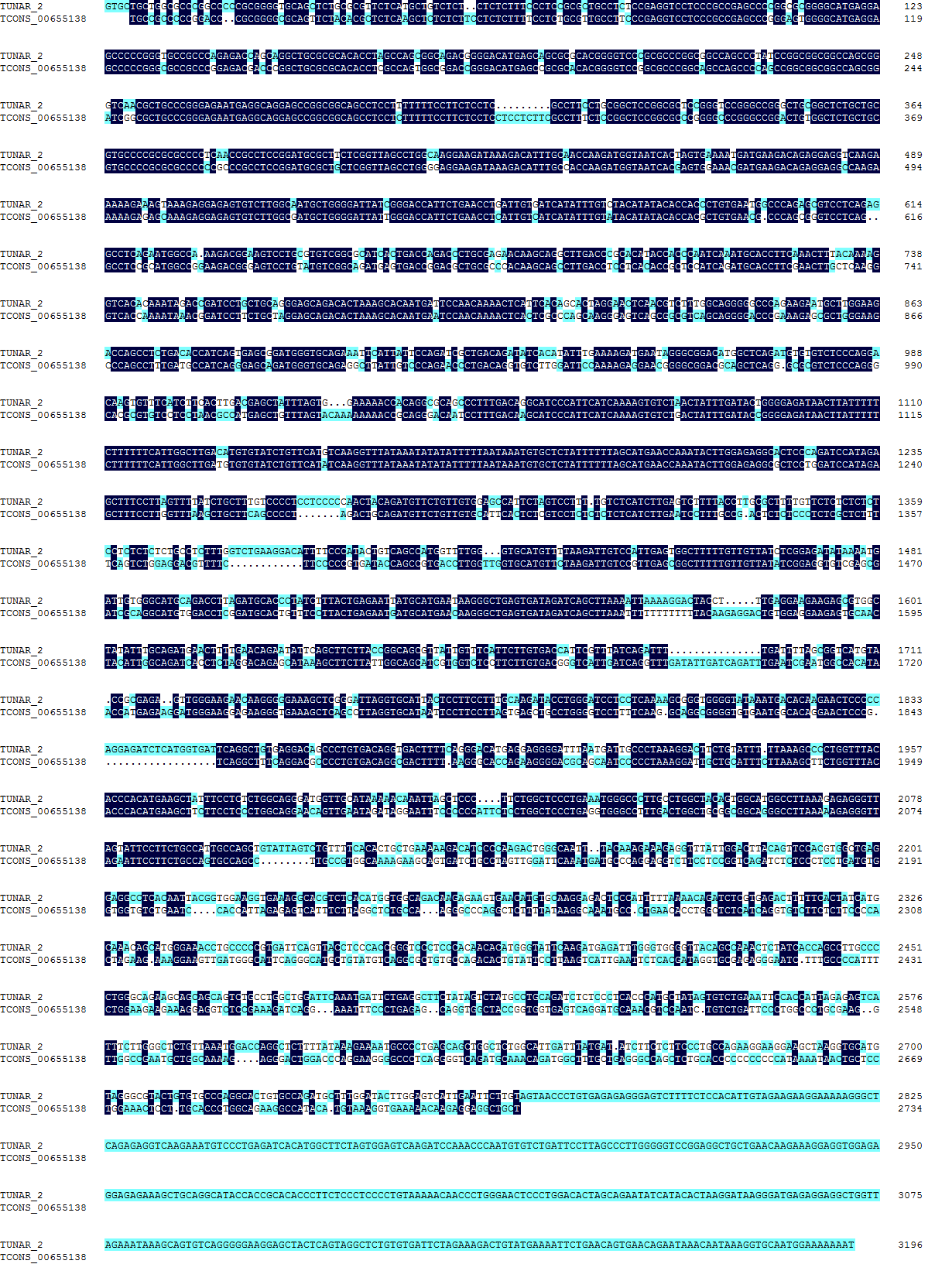


**Figure S14.** Alignment results of TCONS_00655138 with human *TUNAR* gene.
